# Binning enables efficient host genome reconstruction in cnidarian holobionts

**DOI:** 10.1093/gigascience/giy075

**Published:** 2018-07-18

**Authors:** Juan Sebastián Celis, Daniel Wibberg, Catalina Ramírez-Portilla, Oliver Rupp, Alexander Sczyrba, Anika Winkler, Jörn Kalinowski, Thomas Wilke

**Affiliations:** 1 Animal Ecology and Systematics, Justus Liebig University Giessen. Heinrich-Buff-Ring 26–32 (IFZ), 35392 Giessen, Germany; 2 Corporation Center of Excellence in Marine Sciences, Cra 54 No 106–18, Bogotá, Colombia; 3 Center for Biotechnology, Bielefeld University, Universitätsstraße 27, 33615 Bielefeld, Germany; 4 Evolutionary Biology and Ecology, Université libre de Bruxelles, Av. Franklin D. Roosevelt 50, CP 160/12, B-1050 Brussels, Belgium; 5 Bioinformatics and Systems Biology, Justus Liebig University Giessen, Heinrich-Buff-Ring 58, 35392 Giessen, Germany

**Keywords:** hologenome, holobiont, binning, high-throughput sequencing, *Porites rus*, Scleractinia

## Abstract

**Background:**

Many cnidarians, including stony corals, engage in complex symbiotic associations, comprising the eukaryotic host, photosynthetic algae, and highly diverse microbial communities—together referred to as holobiont. This taxonomic complexity makes sequencing and assembling coral host genomes extremely challenging. Therefore, previous cnidarian genomic projects were based on symbiont-free tissue samples. However, this approach may not be applicable to the majority of cnidarian species for ecological reasons. We therefore evaluated the performance of an alternative method based on sequence binning for reconstructing the genome of the stony coral *Porites rus* from a hologenomic sample and compared it to traditional approaches.

**Results:**

Our results demonstrate that binning performs well for hologenomic data, producing sufficient reads for assembling the draft genome of *P. rus*. An assembly evaluation based on operational criteria showed results that were comparable to symbiont-free approaches in terms of completeness and usefulness, despite a high degree of fragmentation in our assembly. In addition, we found that binning provides sufficient data for exploratory *k*-mer estimation of genomic features, such as genome size and heterozygosity.

**Conclusions:**

Binning constitutes a powerful approach for disentangling taxonomically complex coral hologenomes. Considering the recent decline of coral reefs on the one hand and previous limitations to coral genome sequencing on the other hand, binning may facilitate rapid and reliable genome assembly. This study also provides an important milestone in advancing binning from the metagenomic to the hologenomic and from the prokaryotic to the eukaryotic level.

## Background

Symbiotic associations are common across the tree of life, being fundamental to nearly all aspects of host function and fitness [1]. The ecological and evolutionary implications of symbiotic relationships [2–4] have promoted the “holobiont” concept, which regards these assemblages as integrated biomolecular networks of the host and its associated microorganisms [5]. The concept has also been extended to the genomic level of the organisms, where the host genome along with the individual genomes of the symbiotic partners constitute a “hologenome” [1, 6]. Hence, this biological entity might represent a level of organization at which natural selection operates [1].

Well-known examples of symbiotic relationships can be found in reef-building stony corals (order Scleractinia) [7]. The success of these organisms in building large three-dimensional carbonate structures mainly depends on a close association of the cnidarian host and photosynthetic dinoflagellates (zooxanthellae; *Symbiodinium* spp.). The latter provide up to 90% of the energy requirements of the coral [8]. Furthermore, a highly complex synergistic interdependence with bacteria [7, 9], endolithic algae [10], fungi [9, 11], archaea [12], and viruses [13, 14] has been uncovered.

The complexity of the symbiotic assemblage makes sequencing and assembly of coral genomes extremely challenging. Reasons include the high taxonomic diversity of the hologenome [12], the firm integration of most endosymbionts into host tissues [8, 11, 15], the often high density of *in hospite* symbiont cells [8], and the large size of some symbiont genomes, such as those of zooxanthellae, which may be considerably larger than the coral host genome [16–18]. As a consequence, whole genome-based studies of scleractinian corals (and other holobionts) are still scarce [16, 19]. The low number of reference genomes, in turn, further hampers the assembly of novel genomes and the development of other approaches that rely on the information they contain. Additionally, assembling genomes of diploid organisms with significant levels of heterozygosity poses a challenge that is typically addressed by time-consuming and, in the case of non-model organisms and/or wild-type samples, challenging methods, such as gamete-, inbred line-, or fosmid-based hierarchical sequencing [20].

Due to the holobiontic nature of corals and other cnidarians, most previous genome sequencing projects were based on “symbiont-free” host DNA, i.e., an *in situ* reduction of taxonomic complexity. Respective approaches included the use of gametes (e.g., in the scleractinian coral *Acropora digitifera* [16]), larval stages (e.g., in the sea anemone *Nematostella vectensis* [21]), tissues obtained from aposymbiotic (symbiont-free) specimens (e.g., in the symbiont-facultative sea anemone *Exaiptasia pallida* [22]), and/or using nuclei isolation (e.g., in the corallimorpharians *Amplexidiscus fenestrafer* and *Discosoma* spp. [23]). Whereas these studies have pioneered whole-genome sequencing in cnidarians, the respective approaches might not be applicable to the vast majority of stony coral species for ecological (e.g., most species are zooxanthellate [24], making it difficult to obtain aposymbiotic tissues [25]) and/or physiological reasons (e.g., constraints in reproductive cycles, spawning time, and developmental stages). Moreover, given that the hologenome serves as an integrated network of gene functions with horizontal gene transfer reported for cnidarians [19], symbiont-free DNA from host organisms may only explain one side of molecular evolution. Therefore, a better understanding of the function of the coral holobiont requires approaches based on hologenome and holotranscriptome analyses. However, as stated above, the analysis of hologenomic sequences remains challenging mainly due to the complexity of symbiotic assemblages and uneven sequencing coverage of the various taxa involved [26]. This problem might be solved *in silico* with bioinformatics tools such as binning. Originally designed for metagenomic data, binning aims at assigning groups of sequences into defined taxonomic units [27] based on reference genomes [28], differential sequence coverage data [29], and/or tetra nucleotide frequency information [30, 31]. It thus provides a possibility for reducing the complexity of the data, enabling independent genome assembly of the binned contigs [27]. However, binning remains bioinformatically challenging, as highly complex metagenomes may potentially lead to inaccurate binning and consequently undesirable assembly results [32]. In corals, binning has recently been used for determining the microbial community profile and assembling the genomes of specific bacterial groups (e.g., *Endozoicomonas* spp. [33]). However, to date, it has not been applied to eukaryotic host genomes within a complex holobiont.

**Figure 1:**
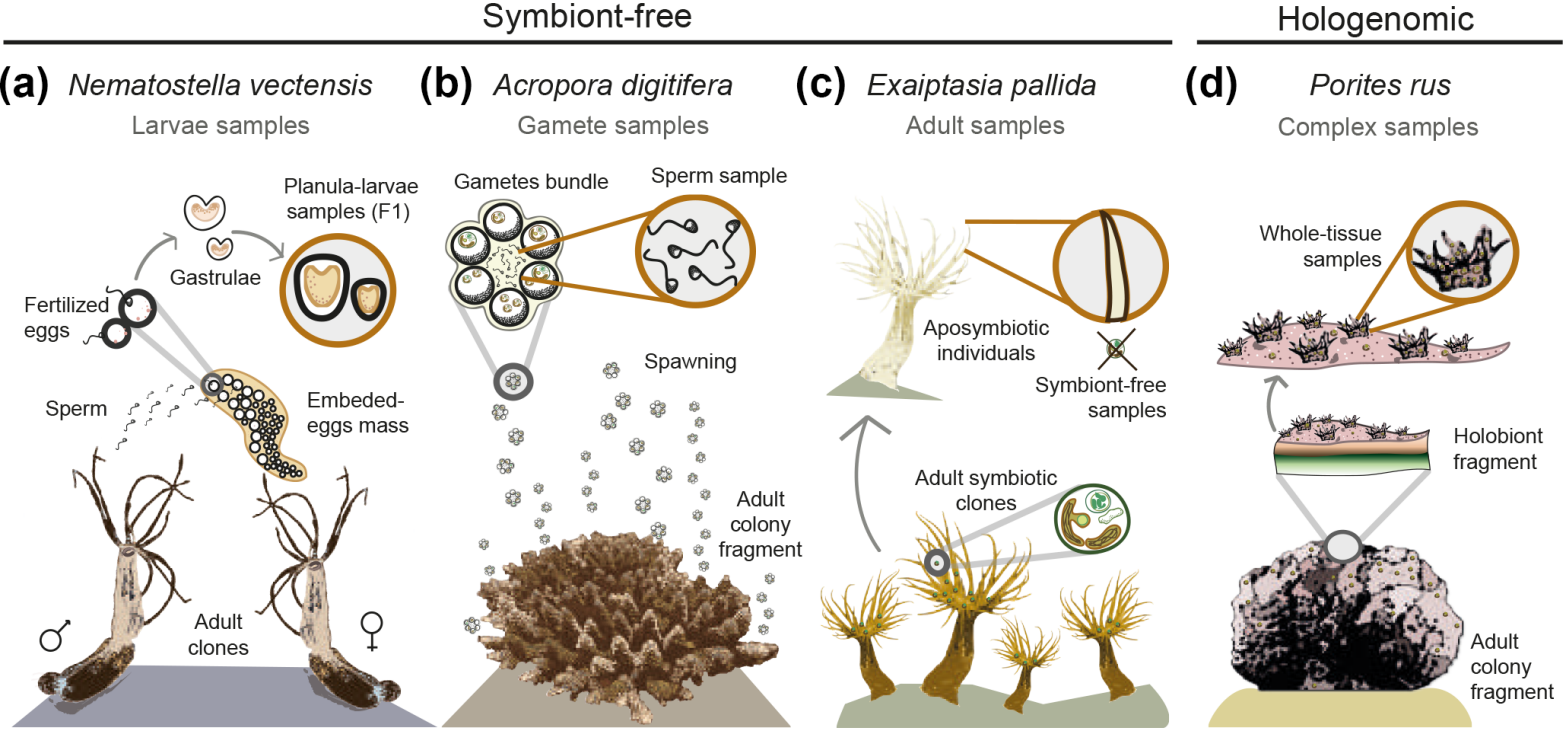
Host DNA sampling approaches in cnidarian genomic projects. **(a)** Use of first-generation (F1) larvae, as in the sea anemone *N. vectensis* [21]. **(b)** Use of sperm from a single colony, as in the stone coral *A. digitifera* [16]. **(c)** Inducing an aposymbiotic state in adult clones, as in the sea anemone *E. pallida* [22]. **(d)** Use of holobiont samples with a subsequent binning step, as in the current study.

In this study, we evaluated the performance of binning for reconstructing the host genome of a coral holobiont (*Porites rus*; Scleractinia) as an alternative to prevalent symbiont-free approaches. In the first step, we performed a 16S ribosomal RNA (16S rRNA) gene amplicon analysis in order to assess the *P. rus* hologenome complexity. In the second step, we conducted high-throughput DNA and RNA sequencing (RNA-seq) of a hologenomic sample. The third step included hologenome assembly, binning, and reconstruction of the *P. rus* host genome. In the final step, we compared the quality of our binning-based assembly with assemblies based on traditional, symbiont-free DNA approaches (see Fig.1). To do this, we performed z-score calculations using the operational criteria completeness (e.g., expected gene content), contiguity (e.g., fragmentation degree of the assembly), and usefulness (e.g., proportion of scaffolds that are greater than the length of an average gene in various model organisms).

Taking into account the recent decline of coral reefs and the challenges associated with deciphering the genomes of zooxanthellate corals, our study may lay the foundations for a rapid and reliable genome assembly in these reef-building organisms. Moreover, this study might be of general interest for geneticists and bioinformaticians as it provides an important milestone in taking binning from the metagenomic to the hologenomic and from the prokaryotic to the eukaryotic level.

## Data Description

### Hologenomic DNA/RNA samples

Hologenomic DNA/RNA for subsequent analyses was extracted from an adult *P. rus* colony using standard isolation methods. RNA and DNA samples are available at the University of Giessen Systematics and Biodiversity Collection [34] with voucher numbers 21 615 and 21 614, respectively.

### 16S rRNA-seq data

The 16S rRNA gene amplicon sequencing was performed in order to determine the taxonomic complexity of the hologenome. Accordingly, the hypervariable regions V3 and V4 of the 16S rRNA gene were amplified across taxa. The 16S dataset is available in the Bioproject PRJEB25185.

### Holotranscriptome data

Hologenomic RNA was extracted from an adult *P. rus* colony, and sequencing libraries were prepared from mRNA to aid gene prediction. The European Molecular Biology Laboratory (EMBL)-European Bioinformatics Institute (EBI) Annotare project ID for the holotranscriptome data is E-MTAB-6535.

### Hologenome data

Hologenomic DNA was extracted from an adult *P. rus* adult colony for whole-hologenome sequencing, binning, and coral host genome assembly. The hologenome raw data are available under the Bioproject ID PRJEB23570.

### Assembled genome and transcriptome

The assembled coral host genomes were used to assess the performance of our *in silico* approach for reducing the taxonomic complexity in cnidarians compared to *in situ* approaches. The EMBL-EBI accession numbers are OKRP01000001-OKRP01014982 for the genome. The transcriptome data are available via the Annotare project ID E-MTAB-6535 (Supplementary Table S1).

### Analyses

#### Taxonomic composition of the P. rus hologenome

In total, 439 operational taxonomic units (OTUs) were obtained and taxonomically classified from kingdom to genus level based on 16S rRNA gene amplicons. The majority of reads (∼60%) were assigned to eukaryotic replicons. This included the 16S rRNA gene of the *P. rus* mitochondrial genome (∼38%) and the chloroplast 16S rRNA gene of various algae such as *Synedra* spp. and *Ostreobium* spp. (∼21%). The bacterial superkingdom was represented by ∼38% of the reads (see Supplementary Fig. S1 for a detailed description of 16S rRNA amplicon results). Due to the high representation of *P. rus* genes, the sample was considered suitable for host-genome reconstructions based on binning.

#### General features of the assembled P. rus draft genome

Approximately 473 million reads with 141 Gb sequence information of high-quality sequencing data (mate pair and paired end) were generated. After quality trimming and filtering, the metagenome assembly resulted in  1,829,146 contigs (>100 bp) (N50 = 1.3 kb) with a size of 947 Mb. About 45% of the ∼212 million metagenome reads were mapped back onto these contigs. The resulting bam-file was used for binning with MetaBat. In total, 13 host genome bins were predicted, ranging from 250 kb to 370 Mb. Based on a manual inspection using Basic Local Alignment Search Tool (BLASTx), r2cat, and the reference genomes of *A. digitifera*, *E. pallida*, and *N. vectensis*, all bins were classified as *P. rus*, thus representing its draft genome. Using these genome bins as a reference, ∼148 million reads were assigned to the coral bins through read mapping. These reads were used for a single genome assembly adapted to diploid organisms, resulting in a 470-Mb draft genome (∼72x coverage), comprising 14,982 scaffolds (N50 = 137 kb) and  81,422 contigs (N50 = 5.3 kb) and featuring a guanine-cytosine (GC) content of 38.86% (Table 1; for assembly metrics of the four genomes, see Supplementary Table S2).

**Table 1:** Statistics for the *P. rus* genome assembly

Parameter	Value
Median genome size (*k*-mer = 21 estimation)	404.76 Mb
Total size of genome assembly ( 14,982 scaffolds)	470 Mb
Total contig size ( 81,422 contigs)	332 Mb
Scaffold N50	137 kb
Longest scaffold	1.19 Mb
Contig N50	5.3 kb
Longest contig	65 667 bp
GC (guanine-cytosine) content	38.86%
Number of predicted genes	39 453

### *P. rus* genome annotation

In total , 39,453 protein-coding genes were predicted using the *de novo* gene prediction tool Genemark-ES. RNA-sequencing validation confirmed 9,662 (∼25%) of the predicted gene models, whereas for the remaining predicted genes,  21,865 homologous reference genes were detected in the National Center for Biotechnology Information database (Status November 2017, threshold 1 × 10^−5^). They mainly refer to other coral genomes (e.g., *A. digitifera*) and hence represent potential orthologs. InterProScan identified 31, 611 genes with known functional domains. Additionally,  17,754 of these genes could be assigned to Gene Ontology term numbers. InterPro numbers could be assigned to  25,966 genes and enzyme commission number to 1,191 enzyme-encoding genes.

### *K*-mer distribution and estimation of genomic features

**Figure 2:**
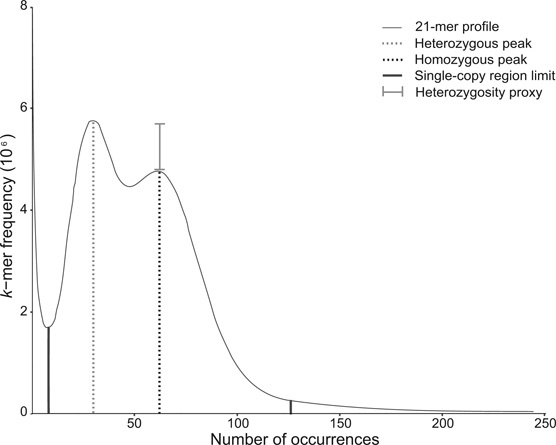
The 21-mer frequency distribution of *P. rus* reads. The profile shows heterozygous and homozygous peaks at ∼32x and ∼63x, respectively, as well as the single-copy region that ends around ∼126x coverage. *K*-mer occurrences of >250x coverage likely correspond to repetitive sequences and < 11x coverage to sequencing errors. The difference in peak heights at 32x and 63x coverage is a proxy for the degree of heterozygosity of the genome.

The 21-mer frequency distribution and the complete read dataset were chosen for performing the final genome size estimation since both coverage peak and size of the repetitive and single-copy regions closely stabilized around this value (Supplementary Fig. S2). Moreover, this *k*-mer size has been suggested as adequate in previous comparative experiments [35]. The *k*-mer frequency displayed a double-peak profile, with the first peak (heterozygous, 32x coverage) taller than the second peak (homozygous, 63x coverage), indicating high heterozygosity [35]. From the total distribution, 1.89% of the sequences likely corresponded to sequencing errors (high frequencies with less than 11x coverage). Therefore, they were discarded prior to downstream analyses. In addition, 18.81% (>250x coverage) of the total *k*-mer occurrences corresponded to repetitive sequences (Fig. 2).

As for the genome size estimations, there were no significant differences when using four *k*-mer sizes (Kruskal-Wallis test, *P* value = 0.29, Supplementary Table S4). In contrast, the three approaches (Waterman, gce, and GenomeScope) yielded significantly different estimations (Kruskal-Wallis test, *P* value = 0.0001), particularly between the Poisson-based approaches (Waterman and gce) and the negative binomial-based GenomeScope (Supplementary Table S5). Using the 21-mer frequency distribution, the Waterman and gce approaches estimated the *P. rus* haploid genome size to be 419.8 Mb and 404.7 Mb, respectively. In contrast, the GenomeScope approach suggested a genome size of about 332.8 Mb. The median genome size estimations obtained from the 21-mer distributions using the complete dataset of *P. rus* reads was 404.76 ± 37.96 Mb (Supplementary Table S5). This value was used for downstream calculations.

### Comparative assessment of the operational criteria completeness, contiguity, and usefulness

**Figure 3:**
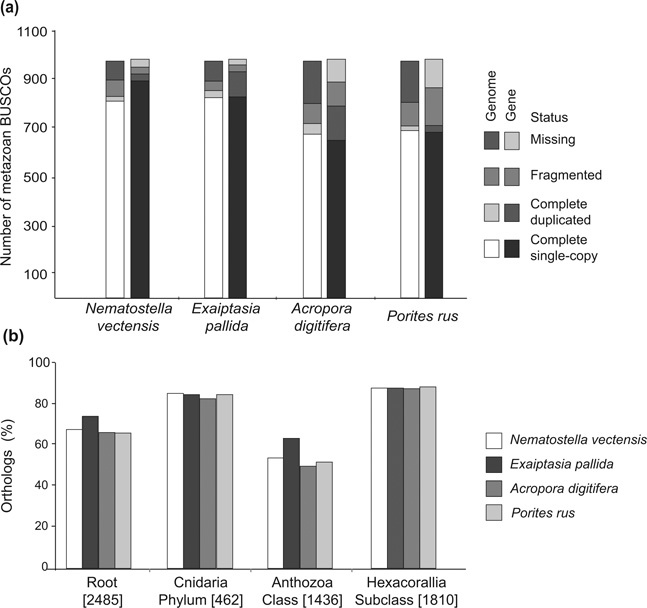
Completeness assessment of four cnidarian genomes. **(a)** Bar plots represent the number of metazoan BUSCOs at the genome and gene dataset levels in the sea anemones *N. vectensis* and *E. pallida* and the stony corals *A. digitifera* and *P. rus*. **(b)** Percentage of orthologs found in each genome assembly at different taxonomic levels. The numbers in square brackets represent the total number of orthologs reported in the original dataset.

We calculated z-scores for six parameters in order to assess the assemblies’ completeness. For the parameters “number of complete single-copy BUSCOs [Benchmarking Universal Single-Copy Orthologs] in the genome” and “number of complete single-copy BUSCOs at gene set levels,” the two sea anemone assemblies (*N. vectensis* and *E. pallida*) performed best, followed by the two stony coral assemblies (*A. digitifera* and *P. rus*) (Supplementary Table S3, Fig. 3a). Similar performance values were inferred for the remaining four completeness parameters (i.e., ortholog groups at root, Cnidaria, Anthozoa, and Hexacorallia levels), although the *P. rus* assembly overperformed in the parameter “ortholog groups at Hexacorallia level” (Supplementary Table S3, Fig. 3b). The sum of z-scores calculated for both the BUSCO and Reef Genomics database showed that the *E. pallida* assembly was superior in terms of completeness, followed by the *N. vectensis*, *P. rus*, and *A. digitifera* assemblies (for detailed z-score information, see Supplementary Table S3).

In order to assess the operational criterion contiguity for the four genome assemblies, we calculated z-scores for seven parameters (see Supplementary Table S3). The *P. rus* assembly did not overperform in any of these parameters and underperformed in the following four of seven parameters: NG50 scaffold length, NG50 contig length, N content, and contigs >10 kb. According to the cumulative z-score analysis for the contiguity metrics, the *A. digitifera* assembly was ranked best, followed by the *E. pallida*, *N. vectensis*, and *P. rus* assemblies (Supplementary Table S3).

**Figure 4:**
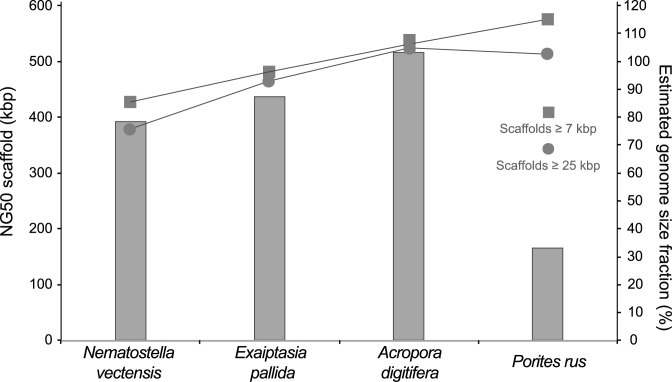
Usefulness assessment based on NG50 scaffold length and fraction of estimated genome sizes represented by gene-sized scaffolds of four cnidarian genome assemblies. NG50 scaffold length (primary *y* axis) is a metric for assessing the contiguity of assemblies, without disregarding differences in the estimated sizes of the genomes. The percentage of the estimated genome size that corresponds to gene-sized scaffolds, according to the average length in invertebrates (≥7 kb) and vertebrates (≥25 kb), are proxies for the usefulness of the assembled sequences (secondary *y* axis).

The final operational criterion, usefulness, was assessed based on the percentage of the estimated genome represented by both vertebrate and invertebrate gene-sized scaffolds. In this study, the average invertebrate gene size was estimated to be 7 kb. Additionally, the previously calculated average size of vertebrate genes (25 kb) [36] was included for evaluating result consistencies and to add a stringency level to the usefulness evaluation of the assemblies. For the parameter “percentage of estimated genome size contained in scaffolds of at least 25 kb,” the *A. digitifera* assembly performed best, and for the parameter “percentage of estimated genome size contained in scaffolds of at least 7 kb,” the *P. rus* assembly performed best (Supplementary Table S3, Fig. 4). According to the cumulative z-score analysis of the criterion usefulness, the *P. rus* and *A. digitifera* assemblies outperformed the *E. pallida* and *N. vectensis* assemblies (Supplementary Table S3).

**Figure 5:**
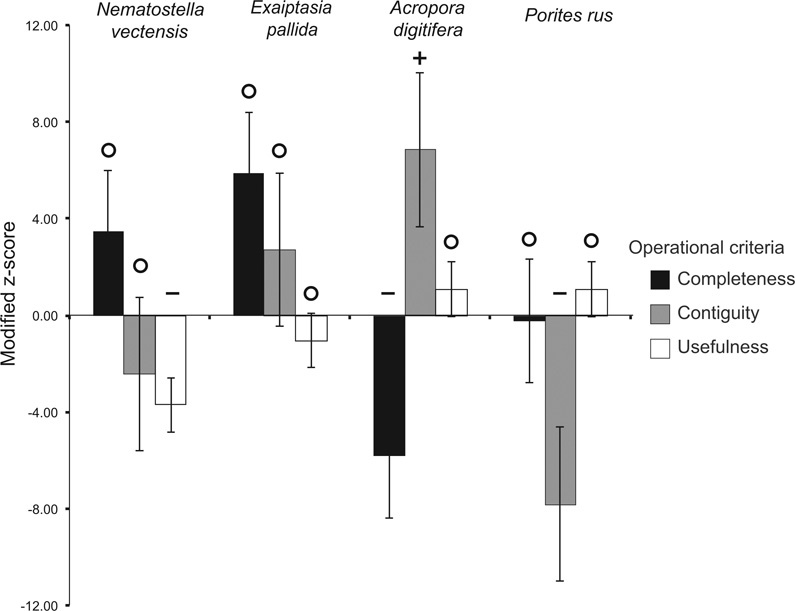
Graphic summary for the assessment of the operational criteria completeness, contiguity, and usefulness in four cnidarian genome assemblies. High z-scores indicate good performance; low scores indicate poor performance. Error bars represent the standard error of the cumulative scores calculated across assemblies. Assembly classifications are based on the standard deviation (SD) of each z-score from the median: + good (≥ 1 SD), O average, —poor (≤ 1 SD).

Overall, the hologenome-based assembly of the *P. rus* genome had superior performance for the criterion usefulness (together with the *A. digitifera* assembly), average performance for the criterion completeness, and inferior performance for the criterion contiguity (Fig.5, Supplementary Table S3).

## Discussion

This study aimed at evaluating the performance of binning, an approach previously used in metagenomics, for facilitating the host genome assembly of a complex coral holobiont. Our results demonstrate that it is a suitable method for extracting coral reads from a hologenomic dataset, allowing for reference-independent reconstruction of the *P. rus* draft genome. A comparison with other cnidarian genome assemblies showed that in spite of the relatively high degree of fragmentation of the *P. rus* assembly informed by contiguity metrics, its completeness and usefulness were of similar or even higher quality than those of traditional symbiont-free assemblies. Additionally, our analyses revealed that coral-binned reads might supply sufficient information for genomic *k*-mer-based estimations of, e.g., degree of heterozygosity, repetitive content, and genome size.

### Binning in coral hologenomes

Bioinformatics tools such as binning may constitute an *in silico* alternative for reducing the complexity of a hologenomic sample. However, until now, binning has mainly been used in metagenomics, including the identification of bacterial communities and the assembly of bacterial genomes in cnidarian holobionts [33]. Here, we demonstrate that binning is effective in reducing the complexity of hologenomic data, facilitating independent analyses of the coral binned reads [27, 37]. Moreover, it works well in cases where reference genomes are scarce, such as in corals. This may be due to the fact that binning is not exclusively based on sequence similarity information but also on other reference-free genomic signatures (including probabilistic distances of tetranucleotide frequencies and contig abundances). Therefore, the grouping of hologenomic reads typically results in highly confident bins [27, 38].

Of interest for hologenomic projects is the fact that binned reads enable the estimation of genomic features (e.g., heterozygosity, repetitive content, and genome size), based on assembly-independent methods such as the *k*-mer-based approach. In fact, from the 21-mer frequency distribution profile obtained (Fig.2), we were able to confirm the high degree of heterozygosity of the diploid target colony used in this study. In addition, we determined the repetitive content of the *P. rus* genome (18.81%), which was similar to those reported for the stony coral *A. digitifera* (13%) [16] and the sea anemones *E. pallida* (26%) [22] and *N. vectensis* (26%) [21]. Moreover, binning provided sufficient reads for testing three *k-*mer-based methods of genome size estimation. Although the Waterman and gce methods are based on the assumption that the *k*-mer frequency profile should approximate a Poisson distribution [39, 40], which was not the case for our double-peak 21-mer distribution, both methods produced values similar to those previously reported for stony corals and sea anemones [22, 41]. These findings suggest that despite the heterozygosity found in the *P. rus* genome, Poisson-based *k*-mer approaches may produce reliable genome size estimations.

In conclusion, coral-binned reads, together with *k*-mer approaches, may produce comprehensive estimations of genomic features such as repeat content, degree of heterozygosity, and genome size. This information, in turn, may help define library types, insert sizes, and sequencing coverage required for sequencing and assembling high-quality genomes [42].

### Comparative quality assessment of host genome assemblies

Comparing our binning-based host genome assembly with assemblies based on symbiont-free DNA, all approaches showed a similar performance in terms of completeness and usefulness (Fig. 5, Supplementary Table S3). In particular, the percentage of scaffolds spanning the average invertebrate gene length in our binning-based assembly was of comparable or even superior quality to that of symbiont-free assemblies. However, the fragmentation of our assembly (as indicated by N50 and NG50 contig lengths; Supplementary Table S2) was considerably higher, making structural genome analyses or other assessments that rely on this feature (e.g., synteny) challenging or even impossible. Therefore, standard contiguity metrics should be interpreted carefully and according to the goals of the respective study. For instance, when aiming at reconstructing multiple sequences that are present in the sample at varying levels of abundance (as previously acknowledged in metagenomics and hologenomics), contiguity could be less important than the criteria completeness and usefulness [43]. Nonetheless, the inclusion of long-read data (e.g., Pacific Biosciences/Nanopore sequencing) could increase binning performance, leading to longer binned contigs. This might greatly reduce the fragmentation of host genome binning-based assemblies.

Finally, as we compared assemblies from different cnidarian species with distinct genome features (e.g., repeat content, number of introns, genome size, and heterozygosity degree), our study did not aim at comparing assembler performances. Rather, we were interested in whether binning can provide sufficient data for assembling host genomes of comparable quality to assemblies based on symbiont-free DNA. Our results demonstrate that binning performs well in reducing hologenomic data complexity aimed at assembling host genomes. However, it is recommended that several assembly strategies be tested in order to improve final assembly contiguity based on the features of the binned data.

In conclusion, our study demonstrate that both symbiont-free and hologenomic binning-based strategies enable host genome reconstruction. The choice of either approach for future coral genome assemblies thus depends on the study taxon (e.g., whether or not symbiont-free DNA can be obtained), the scientific question of interest, and whether the study has a genomic, metagenomic, or hologenomic focus.

### Binning and host genome reconstruction: caveats and suggestions

Despite the high taxonomic complexity of coral holobionts, binning performed well in our hologenomic dataset. However, it is important to note that our hologenomic sample turned out to be rich in coral DNA, which may have contributed to the good performance of our binning approach. At this point, it is difficult to tell whether hologenomic samples with a higher fraction of symbiont-DNA may be equally suited for host genome reconstruction or whether they require a prior enrichment step.

It is also important to note that binning may be sensitive to problems associated with genomic interactions within a holobiont. In particular, horizontal gene transfer between the coral host and the microbial community [19] may reduce binning accuracy, especially in composition-based approaches and underrepresented taxa [27]. A possibility to address this issue is to generate subtraction reference genomic libraries of *Symbiodinium* symbionts, as recently implemented [25]. Alternatively, the number of samples from the target species could be increased because the higher the abundance variation among samples of a target species, the more likely binning tools will produce a reliable genome bin for this species [38]. In addition, there are two inherent challenges associated with binning. First, implementation of this relatively new approach may be complex, requiring a considerable degree of bioinformatics skills. Second, automatic parameter selection based on the underlying data is not yet available. Thus, users have to explore different presets to achieve the best result for their individual datasets [38].

In conclusion, we demonstrate that binning might handle cnidarian hologenomic sequencing samples well, providing enough binned reads for assembling the draft genome of the stony coral *P. rus*. A comparative assessment of the operational criteria contiguity, completeness, and usefulness across cnidarian genome projects revealed that the binning approach displayed comparable results to symbiont-free approaches in terms of completeness and usefulness, despite a high degree of fragmentation. In addition, we demonstrate that binning might supply data for exploratory *k*-mer-based estimations of genomic features such as genome size, heterozygosity, and repetitive content. Thus, binning constitutes a powerful tool for reducing the taxonomic complexity of holobiontic samples. Moreover, even though binning was originally designed for metagenomic analyses, we showed that it is capable of handling eukaryotic data.

However, binning may not *per se* be the first choice for future coral genome projects. Whether to use *in silico* approaches for reducing the taxonomic complexity of holobiontic samples or alternative *in situ* approaches largely depends on the study taxon and the scientific question of interest. Nonetheless, given the methodological challenges in hologenomics on the one hand and the low number of cnidarian host-genome assemblies on the other hand, binning may open the door to rapid and reliable host genome reconstructions in cnidarian holobionts. This in turn might help generate the genomic data necessary for assessing the complex genetic interactions within the coral holobiont and thus the genetic base of coral disease susceptibility and resistance.

### Potential implications

Our study shows that sequence binning is a valid tool for reducing the high taxonomical complexity of a coral holobiont. This *in silico* approach may also be applicable for disentangling other holobiont assemblages, particularly when *in situ* taxonomic complexity reduction is not desired or feasible. Taking into account that eukaryotes such as plants and animals are no longer regarded as isolated entities [1, 5, 44], our results may lay the foundations for a rapid and reliable genome assembly in a broader range of holobionts other than cnidarians. This study thus provides an important milestone in advancing binning from the metagenomic to the hologenomic and from the prokaryotic to the eukaryotic level.

## Methods

### Target colony and tissue sampling

An adult *P. rus* colony was imported from Indonesia in 2007 (CITES permit number 14 846/IV/SATS-LN/2007) and kept in the marine facilities at Justus Liebig University Giessen. The colony was maintained in a seawater system at approximately 26°C on a 10:14 h light–dark cycle. A fragment of ∼9 cm^2^ was taken from the colony for hologenomic DNA/RNA isolation. The tissue was removed by scraping the fragment's surface with a sterilized razor blade as previously recommended [45] and immediately snap-frozen in liquid nitrogen for subsequent analyses. The original *P. rus* colony is in long-term culture at Justus Liebig University Giessen and thus available for further investigation.

### DNA and RNA isolation

High-molecular-weight genomic DNA was isolated from ∼30 mg of tissue using the DNeasy Blood and Tissue Kit (Qiagen, Hilden, Germany) according to the manufacturer's instructions. DNA quality and integrity were assessed with a Nanodrop 2000 photometer (Thermo Fisher Scientific, Waltham, MA, USA) and through visual inspection on a 2% agarose gel. Exact quantity was determined using the Quant-iT PicoGreen dsDNA Assay Kit (Invitrogen, Carlsbad, CA, USA). Total RNA was isolated by incubating 30 mg of tissue with 1 mL TRIzol (Invitrogen) in a 2 -mL tube for 1 hour. Further tissue disruption and homogenization was performed in three 1-minute cycles with grinding beads using a TissueLyser II (Qiagen). RNA was purified from the homogenized sample with Direct-Zol RNA minipreps (Zymo, Irvine, CA, USA) following the manufacturer's protocol. Quantity and quality were assessed using a RNA Pico chip on a Bioanalyzer 2100 (Agilent Technologies, Santa Clara, CA, USA).

### High-throughput 16S rRNA gene amplicon sequencing and processing

In order to determine the taxonomic complexity of the hologenome, high-throughput 16S rRNA gene amplicon sequencing was performed as described by Maus et al. [46]. Primers Pro341F (5΄-CCTACGGGGNBGCASCAG-3΄) and Pro805R (5΄-GACTACNVGGGTATCTAATCC-3΄) [47] were used to amplify the hypervariable regions V3 and V4 of the 16S rRNA gene in Bacteria and Archaea as well as in single-celled algae and other eukaryotes. Multiplex identifier tags and Illumina-specific sequencing adaptors were used for two polymerase chain reaction (PCR) steps. The PCR products, featuring a length of 460 bp, were purified using AMPureXP magnetic beads (Beckman Coulter, Brea, CA, USA). Quality and quantity assessments of 16S rRNA gene amplicons were done using the Agilent 2100 Bioanalyzer system. Then, amplicons were pooled in equimolar amounts for subsequent Illumina MiSeq sequencing (Illumina, San Diego, CA, USA), applying the paired-end protocol. Adapter and primer trimming were performed through an in-house pipeline [48]. For amplicon processing, a pipeline including FLASH v.1.2.11 [49], USEARCH v.8.1 [50], UPARSE v.10.0.240 [51], and the Ribosomal Database Project (RDP) classifier v.2.9 [52] was used as described recently [46, 53, 54]. All sequences that were not merged by FLASH using default settings were filtered out. In addition, sequences with >1 N (ambiguous base) in the sequence read and expected errors >0.5 were also discarded. Resulting data were processed; and OTUs were clustered using USEARCH and taxonomically classified with the RDP classifier in 16S modus. Only hits featuring a confidence value >0.8 were considered. Finally, obtained raw sequence reads were mapped back onto the OTU sequences in order to get quantitative assignments.

### Whole hologenome sequencing, binning, assembly, and annotation of the *P. rus* genome

To obtain hologenome sequence data, a whole-genome-shotgun PCR-free (TruSeq PCR-free DNA Sample Prep Kit; Illumina) and 10-kb mate-pair libraries (Nextera Mate Pair Sample Preparation Kit; Illumina) were generated based on the manufacturer's protocols. Both libraries were sequenced in two HiSeq 1500 rapid runs in paired-end mode (2 × 250 bp). After sequencing and processing of the raw data with Trimmomatic v.0.32 [55] and an in-house pipeline based on CASAVA v.1.8.2. (Illumina) [48], a *de novo* metagenome assembly was performed using the Ray Meta assembler v.2.3.0 [56] with a *k*-mer size of 41 and default settings. All processed raw reads were aligned to the assembled metagenome contigs using Bowtie v.2.2.4 [57]. The resulting bam file was sorted, and read mapping statistics were calculated by applying bamtools v.2.4.1 [58]. To sort the metagenome contigs into genome bins, MetaBAT v.0.21.3 [38] was used by applying default settings. Here, it combines the formation of the tetra-nucleotide frequency and the contig abundance probabilities in order to produce high-quality genome bins out of a large hologenomic dataset. Coral raw reads were extracted by means of read mapping to all coral bins and later reassembled using the gsAssembler v.2.8 (Roche Diagnostics, Mannheim, Germany), applying the heterozygote mode with default settings. Gene prediction in the reconstructed *P. rus* draft genome was done with GeneMark v.4.3.2. [59]. The functional annotation of predicted genes was conducted within the GenDBE annotation platform for eukaryotes [60]. To assess the quality of gene prediction, RNA-seq data were mapped onto the genome sequences. Results were compared manually and using bioinformatics approaches (e.g., bedtools [61]) to the gene prediction results. Due to the lack of high-quality functional annotations of coral genes in public databases, a homology-based assignment of gene functions is not suitable. Therefore, only an annotation describing the structure of the genes based on functional domains was performed with InterProScan [62].

### Sequencing of complementary DNA libraries

In total, 2.25 μg of RNA were used for library preparation with the TruSeq Stranded mRNA Sample Preparation Kit (Illumina). Sequencing of the complementary DNA library was carried out on the Illumina HiSeq 1500 platform following a modified protocol of Verwaaijen et al. [63]. The sample was paired-end sequenced in a rapid run with 2 × 75 bp cycles. Base calling and data processing were accomplished using in-house software (see above). Obtained reads were quality filtered (>Q30) by applying the FASTX tool kit [64]. The dataset was mapped onto the established *P. rus* genome assembly using tophat v.2.1.1 [65]. Two mismatches were allowed to account for possible sequencing errors and allelic variants of the diploid *P. rus* genome. The sequence read analysis platform ReadXplorer v.2.2.3. [66, 67] was used for visualization and further analysis of the data. Reads per kilobase per million reads values were calculated from exported read count tables using the *single best match* option for each library. In addition, gene prediction results were confirmed with ReadXplorer. The RNA-seq and the draft genome data are available on the EBI Annotare server (project ID, E-MTAB-6535) and in European Nucleotide Archive (accession numbers, OKRP01000001-OKRP01014982), respectively.

### *K*-mer-based genomic features estimation and assembly quality assessment

The size of the *P. rus* genome was estimated through short-sequence substring (*k*-mers) frequencies of the reads used for the assembly. Different *k*-mer sizes were implemented, ranging from 17 to 25 bp. Their *k*-mer count distributions were calculated with Jellyfish v.2.0 [68]. In order to account for the lack of cytometric measures for *P. rus* [69] and the highly variable estimations reported for cnidarians [22, 41], these profile distributions were used to perform three independent genome size estimations (see Supplementary Methods for detailed information about genome size estimations). The first approach corresponded to the Waterman estimation method [70], which is based on the relationship between the number of used bases and *k*-mers obtained. The second approach implemented the heterozygous mode of the gce program v.1.0 [39] that, in addition to Waterman's estimation, takes sequencing errors and coverage bias in the *k*-mer distribution into account. The third approach utilized the GenomeScope web interface tool v.1.0. [35], which uses a negative binomial mixture model to account for the genomic complexity of *k*-mer frequency profiles. In addition, the latter approach measures the relative abundances of heterozygous, homozygous, unique, and two-copy sequences. The performance of these three approaches was evaluated based on paired-end reads from the two genomic datasets for which flow cytometry estimations are available (i.e., *A. digitifera* [16] with an estimated haploid size of ∼420 Mb and *E. pallida* [22] with an estimated haploid size of ∼260 Mb ). As these analyses indicated that there is no single superior methodology for coral genome size estimations, we calculated the median genome size based on the 21-mer distribution in the three approaches conducted (see Supplementary methods for detailed description).

Standard statistics for assessing the assembly's contiguity were calculated using QUAST v. 4.4 [71] and the *assemblathon_stats.pl* script [36]. Completeness was estimated by calculating the content of metazoan BUSCOs (e-value: 0.001, dataset v.3.0.2) [72] in the genome and the gene datasets and by determining the presence of orthologs previously described within a comparative genomic framework of reef cnidarians at various taxonomic levels (Reef Genomics database [19]) using BLASTx (e-value: 0.001, blast v.2.2.30 [73]). Finally, the usefulness was estimated by the percentage of genome size in scaffolds higher or equal to the average gene size of invertebrate model organisms, following Bradnam et al. [36] and using the latest protein-coding annotations of the Ensembl 89 Genes dataset [74] for *Caenorhabditis elegans* (WBcel235), *Ciona intestinalis* (KH), *Ciona savignyi* (CSAV 2.0), *Drosophila melanogaster* (BDGP6), and *Saccharomyces cerevisiae* (R64–1-1).

### Comparative quality assessment of binning-based and symbiont-free-based cnidarian assemblies

In order to assess the quality of our hologenome binning-based assembly, we compared it to literature data from the symbiont-free-based genome assemblies of *N. vectensis* v.1.0 [21], *A. digitifera* v.1.1 [16, 75], and *E. pallida* v.1.1 [22] (Supplementary Tables S1 and S2). The comparison was based on the operational criteria completeness with six parameters, contiguity with seven parameters, and usefulness with two parameters (see Supplementary Table S3 for parameter details). Modified z-scores (*sensu* Bradnam et al. [36]) were calculated individually for the respective parameters (Supplementary Table S3). Then, the individual scores were summed up and the standard deviation from the median value was used to assign the quality levels good (+), average (O), and poor (-) to each assembly.

## Supplementary Material

GIGA-D-18-00072.pdf

Response_to_Reviewer_Comments_Original_Submission.pdf

Reviewer_1_Report_(Original_Submission) -- Beate Slaby 04/19/2018 Reviewed

Reviewer_2_Report_(Original_Submission) -- Thomas Hackl 04/20/2018 Reviewed

Additional Files

## Availability of supporting data

New sequence data used in this here has been submitted to the European Bioinformatics Institute (EMBL-EBI) database under project IDs E-MTAB-6535, PRJEB23570, and PRJEB25185 and accession numbers OKRP01000001-OKRP01014982. Additional supporting data is available in the *GigaScience* repository, GigaDB [76].

## Additional files

**Figure S1:** Krona chart depicting the 16S rRNA gene amplicon taxonomical profile.

**Figure S2:** Genome size estimation for *Porites rus* using *k*-mer based approaches.

**Table S1:** *Porites rus*, *Acropora digitifera*, *Nematostella vectensis*, and *Exaiptasia pallida* genome accession numbers.

**Table S2:** Summary genome assemblies’ statistics.

**Table S3:** Summary operational criteria completeness, contiguity, and usefulness.

**Table S4:** *Porites rus k*-mer statistics.

**Table S5:** Summary *k*-mer based genome size estimation.

**Supplemental methods:** Detailed description of the *k*-mer based genome size estimation.

## Abbreviations

16S rRNA: 16S ribosomal RNA; BLAST: Basic local alignment search tool; BUSCO: Benchmarking Universal Single-Copy Ortholog; EMBL-EBI: European Molecular Biology Laboratory; European Bioinformatics Institute; OUT: operational taxonomic unit; PCR: polymerase chain reaction; RDP:Ribosomal Database Project ; RNA-seq: RNA sequencing.
