# Supplementary material for: Binning enables efficient host genome reconstruction in cnidarian holobionts
Source: Gigascience. 2018 Jul 18;7(7):giy075. doi: 10.1093/gigascience/giy075 (PMC6049006; doi:10.1093/gigascience/giy075)
Supplement: GIGA-D-18-00072.pdf [file giy075_giga-d-18-00072.pdf]

GigaScience

Binning Enables Efficient Host Genome Reconstruction in Cnidarian Holobionts

--Manuscript Draft--

**Manuscript Number:**	GIGA-D-18-00072
**Full Title:**	Binning Enables Efficient Host Genome Reconstruction in Cnidarian Holobionts
**Article Type:**	Research
**Funding Information:**	Deutscher Akademischer Austauschdienst (91559856) — Dr. Juan Sebastian Celis<br>Bundesministerium für Bildung und Forschung (031A533) — Not applicable
**Abstract:**	Background: Many cnidarians, including stony corals, engage in complex symbiotic associations, comprising the eukaryotic host, photosynthetic algae, and highly diverse microbial communities - together referred to as holobiont. This taxonomic complexity makes sequencing and assembling coral host genomes extremely challenging. Therefore, previous cnidarian genomic projects were based on symbiont-free tissue samples. However, this approach may not be applicable to the majority of cnidarian species for ecological reasons. We therefore evaluated the performance of an alternative method based on sequence binning for reconstructing the genome of the stony coral Porites rus from a hologenomic sample, and compared it to traditional approaches.<br>Results: Our results demonstrate that binning performs well for hologenomic data, producing sufficient reads for assembling the draft genome of P. rus. An assembly evaluation based on operational criteria showed comparable results to symbiont-free approaches in terms of completeness and usefulness, despite a high degree of fragmentation in our assembly. In addition, we found that binning provides sufficient data for exploratory k-mer estimation of genomic features, such as genome size and heterozygosity.<br>Conclusions: Binning constitutes a powerful approach for disentangling taxonomically complex coral hologenomes. Considering the recent decline of coral reefs on the one hand and previous limitations to coral genome sequencing on the other hand, binning may facilitate rapid and reliable genome assembly. This study also provides an important milestone in advancing binning from the metagenomic to the hologenomic and from the prokaryotic to the eukaryotic level.
**Corresponding Author:**	Juan Sebastian Celis<br>Justus Liebig Universitat Giessen<br>Giessen, Hessen GERMANY
**Corresponding Author Secondary Information:**	
**Corresponding Author's Institution:**	Justus Liebig Universitat Giessen
**Corresponding Author's Secondary Institution:**	
**First Author:**	Juan Sebastian Celis
**First Author Secondary Information:**	
**Order of Authors:**	Juan Sebastian Celis<br>Daniel Wibberg<br>Catalina Ramírez-Portilla<br>Oliver Rupp<br>Alexander Sczyrba<br>Anika Winkler<br>Jörn Kalinowski

Powered by Editorial Manager® and ProduXion Manager® from Aries Systems Corporation

	Thomas Wilke
**Order of Authors Secondary Information:**	
**Opposed Reviewers:**	
**Additional Information:**	
**Question**	**Response**
Are you submitting this manuscript to a special series or article collection?	No
**Experimental design and statistics**<br><br>Full details of the experimental design and statistical methods used should be given in the Methods section, as detailed in our Minimum Standards Reporting Checklist. Information essential to interpreting the data presented should be made available in the figure legends.<br><br>Have you included all the information requested in your manuscript?	Yes
**Resources**<br><br>A description of all resources used, including antibodies, cell lines, animals and software tools, with enough information to allow them to be uniquely identified, should be included in the Methods section. Authors are strongly encouraged to cite Research Resource Identifiers (RRIDs) for antibodies, model organisms and tools, where possible.<br><br>Have you included the information requested as detailed in our Minimum Standards Reporting Checklist?	Yes
**Availability of data and materials**<br><br>All datasets and code on which the conclusions of the paper rely must be either included in your submission or deposited in publicly available repositories (where available and ethically appropriate), referencing such data using a unique identifier in the references and in the "Availability of Data and Materials" section of your manuscript.<br><br>Have you have met the above requirement as detailed in our Minimum Standards Reporting Checklist?	Yes

Powered by Editorial Manager® and ProduXion Manager® from Aries Systems Corporation

Click here to download Manuscript Celis et al, 2018_GigaScience.doc

Binning Enables Efficient Host Genome Reconstruction in Cnidarian Holobionts

Juan Sebastián Celis[1,2*], **Daniel Wibberg**[3], **Catalina Ramírez-Portilla**[1,4], **Oliver Rupp**[5], **Alexander Sczyrba**[3], **Anika Winkler**[3], **Jörn Kalinowski**[3] **and Thomas Wilke**[1,2]

[1]Animal Ecology and Systematics, Justus Liebig University Giessen. Heinrich-Buff-Ring 26-32 (IFZ), 35392 Giessen, Germany. [2]Corporation Center of Excellence in Marine Sciences (CEMarin). Cra 54 No 106-18, Bogotá, Colombia. [3]Center for Biotechnology (CeBiTec), Bielefeld University. Universitätsstraße 27, 33615 Bielefeld, Germany. [4]Evolutionary Biology and Ecology. Université libre de Bruxelles. Av. Franklin D. Roosevelt 50, CP 160/12, B-1050 Brussels, Belgium. [5]Bioinformatics and Systems Biology. Justus Liebig University Giessen. Heinrich-Buff-Ring 58, 35392 Giessen, Germany.

JSC*: juan.s.celis-melo@bio.uni-giessen.de

DW: dwibberg@cebitec.uni-bielefeld.de

CRP: catalina.ramirez.portilla@ulb.ac.be

OR: oliver.rupp@computational.bio.uni-giessen.de

AS: asczyrba@cebitec.uni-bielefeld.de

AW: awinkler@cebitec.uni-bielefeld.de

JK: joern@cebitec.uni-bielefeld.de

TW: tom.wilke@allzool.bio.uni-giessen.de

*Corresponding author

Abstract

Background: Many cnidarians, including stony corals, engage in complex symbiotic associations, comprising the eukaryotic host, photosynthetic algae, and highly diverse microbial communities – together referred to as holobiont. This taxonomic complexity makes sequencing and assembling coral host genomes extremely challenging. Therefore, previous cnidarian genomic projects were based on symbiont-free tissue samples. However, this approach may not be applicable to the majority of cnidarian species for ecological reasons. We therefore evaluated the performance of an alternative method based on sequence binning for reconstructing the genome of the stony coral *Porites rus* from a hologenomic sample, and compared it to traditional approaches.

Results: Our results demonstrate that binning performs well for hologenomic data, producing sufficient reads for assembling the draft genome of *P. rus*. An assembly evaluation based on operational criteria showed comparable results to symbiont-free approaches in terms of completeness and usefulness, despite a high degree of fragmentation in our assembly. In addition, we found that binning provides sufficient data for exploratory *k*-mer estimation of genomic features, such as genome size and heterozygosity.

Conclusions: Binning constitutes a powerful approach for disentangling taxonomically complex coral hologenomes. Considering the recent decline of coral reefs on the one hand and previous limitations to coral genome sequencing on the other hand, binning may facilitate rapid and reliable genome assembly. This study also provides an important milestone in advancing binning from the metagenomic to the hologenomic and from the prokaryotic to the eukaryotic level.

Key words: Hologenome, Holobiont, Binning, High-throughput sequencing, *Porites rus*, Scleractinia.

Background

Symbiotic associations are common across the tree of life, being fundamental to nearly all aspects of host function and fitness [1]. The ecological and evolutionary implications of symbiotic relationships [2–4] have promoted the 'holobiont' concept, which regards these assemblages as integrated biomolecular networks of the host and its associated microorganisms [5]. The concept has also been extended to the genomic level of the organisms, where the host genome along with the individual genomes of the symbiotic partners constitute a 'hologenome' [1,6]. Hence, this biological entity might represent a level of organization at which natural selection operates [1].

Well-known examples of symbiotic relationships can be found in reef-building stony corals (order Scleractinia) [7]. The success of these organisms in building large 3-D carbonate structures mainly depends on a close association of the cnidarian host and photosynthetic dinoflagellates (zooxanthellae; *Symbiodinium* spp.). The latter provide up to 90% of the energy requirements of the coral [8]. Furthermore, a highly complex synergistic interdependence with bacteria [7,9], endolithic algae [10], fungi [9,11], archaea [12], and viruses [13,14] has been uncovered.

The complexity of the symbiotic assemblage makes sequencing and assembly of coral genomes extremely challenging. Reasons include i) the high taxonomic diversity of the hologenome [12], ii) the firm integration of most endosymbionts into host tissues [8,11,15], iii) the often high density of *in hospite* symbiont cells [8], and iv) the large size of some symbiont genomes, such as those of zooxanthellae, which may be considerably larger than the coral host genome [16–18]. As a consequence, whole genome-based studies of scleractinian corals (and other holobionts) are still scarce [16,19]. The low number of reference genomes, in turn, further hampers the assembly of novel genomes and the development of other approaches that rely on the information they contain. Additionally, assembling genomes of diploid organisms with significant levels of heterozygosity poses a challenge that is typically addressed by time-consuming and, in the case of non-model

organisms and/or wild-type samples, challenging methods; such as gamete-, inbred line-, or fosmid-based hierarchical sequencing [20].

Due to the holobiontic nature of corals and other cnidarians, most previous genome sequencing projects were based on 'symbiont-free' host DNA, i.e. an *in situ* reduction of taxonomic complexity. Respective approaches included the use of gametes (e.g., in the scleractinian coral *Acropora digitifera* [16]), larval stages (e.g., in the sea anemone *Nematostella vectensis* [21]), tissues obtained from aposymbiotic (symbiont-free) specimens (e.g., in the symbiont-facultative sea anemone *Exaiptasia pallida* [22]), and/or using nuclei isolation (e.g., in the corallimorpharians *Amplexidiscus fenestrafer* and *Discosoma* spp. [23]). Whereas these studies have pioneered whole-genome sequencing in cnidarians, the respective approaches might not be applicable to the vast majority of stony coral species for ecological (e.g., most species are zooxanthellate [24], making it difficult to obtain aposymbiotic tissues [25]) and/or physiological reasons (e.g., constraints in reproductive cycles, spawning time and developmental stages). Moreover, given that the hologenome serves as an integrated network of gene functions with horizontal gene transfer reported for cnidarians [19], symbiont-free DNA from host organisms may only tell one side of molecular evolution. Therefore, a better understanding of the function of the coral holobiont requires approaches based on hologenome and holotranscriptome analyses. However, as stated above the analysis of hologenomic sequences remains challenging mainly due to the complexity of symbiotic assemblages and uneven sequencing coverage of the various taxa involved [26]. This problem might be solved *in silico* with bioinformatics tools such as binning. Originally designed for metagenomic data, it aims at assigning groups of sequences into defined taxonomic units [27] based on reference genomes [28], differential sequence coverage data [29] and/or tetra nucleotide frequency information [30,31]. It thus provides a possibility for reducing the complexity of the data, enabling independent genome assembly of the binned contigs [27]. However, binning remains bioinformatically challenging, as highly complex metagenomes may potentially lead to inaccurate binning and consequently undesirable assembly

results [32]. In corals, binning has recently been used for determining the microbial community profile and assembling the genomes of specific bacterial groups (e.g., *Endozoicomonas* spp. [33]). However, so far it has not been applied to eukaryotic host genomes within a complex holobiont.

In this study, we evaluated the performance of binning for reconstructing the host genome of a coral holobiont (*Porites rus*; Scleractinia) as an alternative to prevalent symbiont-free approaches. In a first step, we performed a 16S rRNA gene amplicon analysis in order to assess the *P. rus* hologenome complexity. In a second step, we conducted high-throughput DNA and RNA sequencing of a hologenomic sample. A third step included hologenome assembly, binning and reconstruction of the *P. rus* host genome. In a final step, we compared the quality of our binning-based assembly with assemblies based on traditional, symbiont-free DNA approaches (see Figure 1). For doing so, we performed z-score calculations using the operational criteria completeness (e.g., expected gene content), contiguity (e.g., fragmentation degree of the assembly), and usefulness (e.g., proportion of scaffolds that are greater than the length of an average gene in various model organisms).

Figure 1. Host DNA sampling approaches in cnidarian genomic projects. a) Use of first generation (F1) larvae, as in the sea anemone *N. vectensis* [21], **b**) use of sperm from a single colony, as in the stone coral *A. digitifera* [16], **c**) inducing an aposymbiotic state in adult clones, as in the sea anemone *E. pallida* [22], and **c**) use of holobiont samples with a subsequent binning step, as in the current study.

Taken into account the recent decline of coral reefs and the challenges associated with deciphering the genomes of zooxanthellate corals, our study may lay the foundations for a rapid and reliable genome assembly in these reef-building organisms. Moreover, this study might be of general interest for geneticists and bioinformaticians as it provides an important milestone in taking binning from the metagenomic to the hologenomic and from the prokaryotic to the eukaryotic level.

Data Description

Hologenomic DNA/RNA samples

Hologenomic DNA/RNA for subsequent analyses was extracted from an adult *Porites rus* colony using standard isolation methods. RNA and DNA samples are available at the University of Giessen Systematics and Biodiversity Collection (UGSB) [34] with voucher numbers 21615 and 21614, respectively.

16S rRNA sequencing data

16S rRNA gene amplicon sequencing was performed in order to determine the taxonomic complexity of the hologenome. Accordingly, the hypervariable regions V3 and V4 of the 16S rRNA gene were amplified across taxa. The 16S dataset is available in the Bioproject PRJEB25185.

Holotranscriptome data

Hologenomic RNA was extracted from an adult *P. rus* colony and sequencing libraries prepared from mRNA to aid gene prediction. The EMBL-EBI Annotare project ID for the holotranscriptome data is E-MTAB-6535.

Hologenome data

Hologenomic DNA was extracted from an adult *P. rus* adult colony for whole-hologenome sequencing, binning and coral host genome assembly. The hologenome raw data is available under the Bioproject ID PRJEB23570.

Assembled genome and transcriptome

The assembled coral host genomes were used to assess the performance of our *in silico* approach for reducing the taxonomic complexity in cnidarians compared to *in situ* approaches. The EMBL-EBI accession numbers are OKRP01000001-OKRP01014982 for the genome. The transcriptome data is available via the Annotare project ID E-MTAB-6535.

Analyses

Taxonomic composition of the *P. rus* hologenome

In total, 439 OTUs were obtained and taxonomically classified from kingdom to genus level based on 16S rRNA gene amplicons. The majority of reads (~60%) was assigned to eukaryotic replicons. This included the 16S rRNA gene of the *P. rus* mitochondrial genome (~38%), and the chloroplast 16S rRNA gene of various algae such as *Synedra* spp. and *Ostreobium* spp. (~21%). The bacterial superkingdom was represented by ~38% of the reads (see Figure S1 for a detailed description of 16S rRNA amplicon results). Due to the high representation of *P. rus* genes, the sample was considered suitable for host-genome reconstructions based on binning.

General features of the assembled *P. rus* draft genome

Approximately 473 million reads with 141 Gb sequence information of high-quality sequencing data (mate-pair and paired end) were generated. After quality trimming and filtering, the metagenome assembly resulted in 1,829,146 contigs (> 100 bp) (N50 = 1.3 kb) with a size of 947 Mb. About 45% of the approx. 212 million metagenome reads were mapped back onto these contigs. The resulting bam-file was used for binning with MetaBat. In total, thirteen host genome bins were predicted, ranging from 250 kbp to 370 Mbp. Based on a manual inspection using *Blastx*, r2cat, and the reference genomes of *A. digitifera*, *E. pallida* and *N. vectensis*, all bins were classified as *P. rus*, thus representing its draft genome. Using these genome bins as reference, approximately 148 million reads were assigned to the coral bins through read mapping. These reads were used for a single

genome assembly adapted to diploid organisms, resulting in a 470 Mbp draft genome (approx. 72x coverage), comprising 14,982 scaffolds (N50 = 137 kbp) and 81,422 contigs (N50 = 5.3 kbp), and featuring a GC-content of 38.86% (Table 1, for assembly metrics of the four genomes see Table S2).

Table 1. Statistics for the *P. rus* genome assembly.

**Parameter**	**Value**
Median genome size (*k*-mer = 21 estimation)	404.76 Mbp
Total size of genome assembly (14982 scaffolds)	470 Mbp
Total contig size (81422 contigs)	332 Mbp
Scaffold N50	137 Kbp
Longest scaffold	1.19 Mbp
Contig N50	5.3 Kbp
Longest contig	65667 bp
GC content	38.86%
Number of predicted genes	39453

P. rus genome annotation

In total 39,453 protein-coding genes were predicted using the *de novo* gene prediction tool Genemark-ES. RNA-seq validation confirmed 9,662 (~25%) of the predicted gene models, whereas for the remaining predicted genes, a total of 21,865 homologous reference genes were detected in the NCBI database (Status November 2017, threshold 1 x 10^{-5}). They mainly refer to other coral genomes (e.g., *A. digitifera*) and hence represent potential orthologs. InterProScan identified 31,611 genes with known functional domains. Additionally, 17,754 of these genes could be assigned to gene ontology (GO) term numbers. InterPro numbers could be assigned to 25,966 genes and EC numbers to 1,191 enzyme-encoding genes.

K-mer distribution and estimation of genomic features

The 21-mer frequency distribution and the complete read dataset were chosen for performing the final genome size estimation since both coverage peak and size of the repetitive and single-copy regions closely stabilized around this value (Figure S2). Moreover, this *k*-mer size has been suggested as adequate in previous comparative experiments [35]. The *k*-mer frequency displayed a double-peak

profile, with the first peak (heterozygous, 32x coverage) taller than the second peak (homozygous, 63x coverage), indicating high heterozygosity [35]. From the total distribution, 1.89% of the sequences likely corresponded to sequencing errors (high frequencies with less than 11x coverage). Therefore, they were discarded prior to downstream analyses. In addition, 18.81% (> 250x coverage) of the total *k*-mer occurrences corresponded to repetitive sequences (Figure 2).

Figure 2. 21-mer frequency distribution of *P. rus* reads. The profile shows heterozygous and homozygous peaks at ~32x and ~63x, respectively, as well as the single-copy region that ends around ~126x coverage. *K*-mer occurrences of > 250x coverage likely correspond to repetitive sequences and < 11x coverage to sequencing errors. The difference in peak heights at 32x and 63x coverage is a proxy for the degree of heterozygosity of the genome.

As for the genome size estimations, there were no significant differences when using four *k*-mer sizes (Kruskal-Wallis test, p-value = 0.29, Table S4). In contrast, the three approaches (Waterman, gce and GenomeScope) yielded significantly different estimations (Kruskal-Wallis test, p-value = 0.0001), particularly between the Poisson based approaches (Waterman's and gce) and the negative binomial-based GenomeScope (Table S5). Using the 21-mer frequency distribution, the Waterman and the gce approaches estimated the *P. rus* haploid genome size to be 419.8 Mbp and 404.7 Mbp, respectively. In contrast, the GenomeScope approach suggested a genome size of about 332.8 Mbp. The median genome size estimations obtained from the 21-mer distributions using the complete dataset of *P. rus* reads was 404.76 ± 37.96 Mbp (Table S5). This value was used for downstream calculations.

Comparative assessment of the operational criteria completeness, contiguity and usefulness

We calculated z-scores for six parameters in order to assess the assemblies' completeness. For the parameters 'number of complete single-copy BUSCOs in the genome' and 'number of complete single-copy BUSCOs at gene set levels', the two sea anemone assemblies (*N. vectensis* and *E. pallida*)

performed best, followed by the two stony coral assemblies (*A. digitifera* and *P. rus*) (Table S3, Figure 3A). Similar performance values were inferred for the remaining four completeness parameters, (i.e., ortholog groups at root, Cnidaria, Anthozoa and Hexacorallia levels), although the *P. rus* assembly overperformed in the parameter 'ortholog groups at Hexacorallia level' (Table S3, Figure 3B). The sum of z-scores calculated for both the BUSCO and Reef Genomics database showed that the *E. pallida* assembly was superior in terms of completeness, followed by the *N. vectensis*, *P. rus* and *A. digitifera* assemblies (for detailed z-score information see Table S3).

Figure 3. Completeness assessment of four cnidarian genomes. a) Bar plots represent the number of metazoan BUSCOs at the genome and gene dataset level in the sea anemones *N. vectensis* and *E. pallida,* and the stony corals *A. digitifera* and *P. rus*. **b**) Percentage of orthologs found in each genome assembly at different taxonomic levels. The numbers in square brackets represent the total number of orthologs reported in the original dataset.

In order to assess the operational criterion contiguity for the four genome assemblies, we calculated z-scores for seven parameters (see Table S3). The *P. rus* assembly did not overperform in any of these parameters and underperformed in four out of seven parameters: NG50 scaffold length, NG50 contig length, N content, and contigs > 10K bp. According to the cumulative z-score analysis for the contiguity metrics, the *A. digitifera* assembly was ranked best, followed by the *E. pallida*, *N. vectensis,* and *P. rus* assemblies (Table S3).

The final operational criterion, usefulness, was assessed based on the percentage of the estimated genome represented by both vertebrate and invertebrate gene-sized scaffolds. In this study, the average invertebrate gene size was estimated to be 7 kbp. Additionally, the previously calculated average size of vertebrate genes (25 kbp) [36] was included for evaluating result consistencies and to add a stringency level to the usefulness evaluation of the assemblies. For the parameter 'percentage of estimated genome size contained in scaffolds of at least 25 kbp', the *A. digitifera* assembly performed best, and for the parameter 'percentage of estimated genome size contained in scaffolds

of at least 25 kbp', the *P. rus* assembly (Table S3, Figure 4). According to the cumulative z-score analysis of the criterion usefulness, the *P. rus* and the *A. digitifera* assemblies outperformed the *E. pallida* and *N. vectensis* assemblies (Table S3).

Figure 4. Usefulness assessment based on NG50 scaffold length and fraction of estimated genome sizes represented by gene-sized scaffolds of four cnidarian genome assemblies. NG50 scaffold length (primary Y axis) is a metric for assessing the contiguity of assemblies, without disregarding differences in the estimated sizes of the genomes. The percentage of the estimated genome size that corresponds to gene size scaffolds, according to the average length in invertebrates (≥ 7 kbp) and vertebrates (≥ 25 kbp), are proxies for the usefulness of the assembled sequences (secondary Y axis).

Overall, the hologenome-based assembly of the *P. rus* genome performed superior for the criterion usefulness (together with the *A. digitifera* assembly), on average for the criterion completeness, and inferior for the criterion contiguity (Figure 5, Table S3).

Figure 5. Graphical summary for the assessment of the operational criteria completeness, contiguity, and usefulness in four cnidarian genome assemblies. High z-scores indicate good performance, low scores poor performance. Error bars represent the standard error of the cumulative scores calculated across assemblies. Assembly classifications are based on the standard deviation (SD) of each z-score from the median: + good (≥ 1SD), O average, - poor (≤ 1SD).

Discussion

This study aimed at evaluating the performance of binning, an approach previously used in metagenomics, for facilitating the host genome assembly of a complex coral holobiont. Our results demonstrated that it is a suitable method for extracting coral reads from a hologenomic dataset, allowing for reference-independent reconstruction of the *Porites rus* draft genome. A comparison with other cnidarian genome assemblies showed that, in spite of the relatively high degree of fragmentation of the *P. rus* assembly informed by contiguity metrics, its completeness and usefulness were of similar or even higher quality than those of traditional symbiont-free assemblies.

Additionally, our analyses revealed that coral-binned reads might supply sufficient information for genomic *k*-mer-based estimations of, for example, degree of heterozygosity, repetitive content, and genome size.

Binning in coral hologenomes

Bioinformatics tools like binning may constitute an *in silico* alternative for reducing the complexity of a hologenomic sample. However, until now binning has mainly been used in metagenomics, including the identification of bacterial communities and the assembly of bacterial genomes in cnidarian holobionts [33]. We here demonstrate that binning is effective in reducing the complexity of hologenomic data, facilitating independent analyses of the coral binned reads [27,37]. Moreover, it works well in cases where reference genomes are scarce, such as in corals. This may be due to the fact that binning is not exclusively based on sequence similarity information but also on other reference-free genomic signatures (including probabilistic distances of tetranucleotide frequencies and contig abundances). Therefore, the grouping of hologenomic reads typically results in highly confident bins [27,38].

Of interest for hologenomic projects is also the fact that binned reads enable the estimation of genomic features (e.g., heterozygosity, repetitive content, and genome size), based on assembly-independent methods such as the *k*-mer based approach. In fact, from the 21-mer frequency distribution profile obtained (Figure 2), we were able to confirm the high degree of heterozygosity of the diploid target colony used in this study. In addition, we determined the repetitive content of the *P. rus* genome (18.81%), which was similar to those reported for the stony coral *A. digitifera* (13%) [16], and the sea anemones *E. pallida* (26%) [22] and *N. vectensis* (26%) [21]. Moreover, binning provided sufficient reads for testing three different *k*-mer-based methods of genome size estimation. Although the Waterman and gce methods are based on the assumption that the *k*-mer frequency profile should approximate a Poisson distribution [39,40], which was not the case for our double-peak 21-mer distribution, both methods produced values similar to those previously reported for

stony corals and sea anemones [22,41]. These findings suggest that, despite the heterozygosity found in the *P. rus* genome, Poisson-based *k*-mer approaches may produce reliable genome size estimations.

In conclusion, coral-binned reads, together with *k*-mer approaches, may produce comprehensive estimations of genomic features like repeat content, degree of heterozygosity, and genome size. This information, in turn, may help defining library types, insert sizes, and sequencing coverage required for sequencing and assembling high-quality genomes [42].

Comparative quality assessment of host genome assemblies

Comparing our binning-based host genome assembly with assemblies based on symbiont-free DNA, all approaches showed a similar performance in terms of completeness and usefulness (Figure 5, Table S3). In particular, the percentage of scaffolds spanning the average invertebrate gene length in our binning-based assembly was of comparable or even superior quality to that of symbiont-free assemblies. However, the fragmentation of our assembly (as indicated by N50 and NG50 contig lengths, Table S2) was considerably higher, making structural genome analyses or other assessments that rely on this feature (e.g., synteny) challenging or even impossible. As a consequence, standard contiguity metrics should be interpreted carefully and according to the goals of the respective study. For instance, when aiming at reconstructing multiple sequences that are present in the sample at varying levels of abundance (as previously acknowledged in metagenomics and hologenomics), contiguity could be less important than the criteria completeness and usefulness [43].

Finally, as we compared assemblies from different cnidarian species with distinct genome features (e.g., repeat content, number of introns, genome size, and heterozygosity degree), our study did not aim at comparing assembler performances. Rather, we were interested in whether binning can provide sufficient data for assembling host genomes of comparable quality to assemblies based on symbiont-free DNA. Our study, indeed, demonstrated that both strategies enable host genome

reconstruction. The choice of methods for future coral genome assemblies thus depends on the study taxon (e.g., whether or not symbiont-free DNA can be obtained), the scientific question of interest, and whether the study has a genomic, metagenomic or hologenomic focus.

Binning and host genome reconstruction: caveats and suggestions

Despite the high taxonomic complexity of coral holobionts, binning performed well in our hologenomic dataset. However, it is important to note that our hologenomic sample turned out to be rich in coral DNA, which may have contributed to the good performance of our binning approach. At this point, it is difficult to tell whether hologenomic samples with a higher fraction of symbiont-DNA may be equally suited for host genome reconstruction or whether they require a prior enrichment step.

It is also important to note that binning may be sensitive to problems associated with genomic interactions within a holobiont. In particular, horizontal gene transfer between the coral host and the microbial community [19] may reduce binning accuracy, especially in composition-based approaches and underrepresented taxa [27]. A possibility to address this issue is to generate subtraction reference genomic libraries of *Symbiodinium* symbionts, as recently implemented [25]. Alternatively, the number of samples from the target species could be increased as the higher the abundance variation among samples of a target species, the more likely binning tools will produce a reliable genome bin for this species [38]. In addition, there are two inherent challenges associated with binning. First, implementation of this relatively new approach may be complex, requiring a considerable degree of bioinformatic skills. Second, automatic parameter selection based on the underlying data is not yet available. Thus, users have to explore different presets to achieve the best result for their individual datasets [38].

In conclusion, we demonstrated that binning may handle cnidarian hologenomic sequencing samples well, providing enough binned reads for assembling the draft genome of the stony coral *P. rus*. A

comparative assessment of the operational criteria contiguity, completeness, and usefulness across cnidarian genome projects revealed that the binning approach displayed comparable results to symbiont-free approaches in terms of completeness and usefulness, despite a high degree of fragmentation. In addition, we demonstrated that binning might supply data for exploratory *k*-mer based estimations of genomic features like genome size, heterozygosity, and repetitive content. Thus, binning constitutes a powerful tool for reducing the taxonomic complexity of holobiontic samples. Moreover, even though binning was originally designed for metagenomic analyses, we show that it is well capable of handling eukaryotic data.

However, binning may not *per se* be the first choice for future coral genome projects. Whether to use *in silico* approaches for reducing the taxonomic complexity of holobiontic samples or alternative *in situ* approaches largely depends on the study taxon and the scientific question of interest. Nonetheless, given the methodological challenges in hologenomics on the one hand and the low number of cnidarian host-genome assemblies on the other hand, binning may open the door to rapid and reliable host genome reconstructions in cnidarian holobionts. This in turn might help generating the genomic data necessary for assessing the complex genetic interactions within the coral holobiont and thus the genetic base of coral disease susceptibility and resistance.

Potential implications

Our study shows that sequence binning is a valid tool for reducing the high taxonomical complexity of a coral holobiont. This *in silico* approach may also be applicable for disentangling other holobiont assemblages, particularly when *in situ* taxonomic complexity reduction is not desired or feasible. Taking into account that eukaryotes such as plants and animal are not longer regarded as isolated entities [1,5,44], our results may lay the foundations for a rapid and reliable genome assembly in a broader range of holobionts other than cnidarians. This study thus provides an important milestone

in advancing binning from the metagenomic to the hologenomic and from the prokaryotic to the eukaryotic level.

Methods

Target colony and tissue sampling

An adult *Porites rus* colony was imported from Indonesia in 2007 (CITES permit number 14846/IV/SATS-LN/2007) and kept in the marine facilities at Justus Liebig University Giessen. The colony was maintained in a seawater system at approximately 26 °C on a 10:14 h light-dark cycle. A fragment of approx. 9 cm^2 was taken from the colony for hologenomic DNA/RNA isolation. The tissue was removed by scraping the fragment´s surface with a sterilized razor blade as previously recommended [45] and immediately snap-frozen in liquid nitrogen for subsequent analyses. The original *P. rus* colony is in long-term culture at Justus Liebig University Giessen and thus available for further investigation.

DNA and RNA isolation

High-molecular-weight genomic DNA (gDNA) was isolated from approx. 30 mg of tissue using the DNeasy Blood and Tissue kit (Qiagen, Hilden, Germany) according to the manufacturer's instructions. DNA quality and integrity were assessed with a Nanodrop 2000 photometer (Thermo Fisher Scientific, Waltham, USA) and through visual inspection on a 2% agarose gel. Exact quantity was determined using the Quant-iT PicoGreen dsDNA Assay Kit (Invitrogen, Carlsbad, USA). Total RNA was isolated by incubating 30 mg of tissue with 1 mL TRIzol (Invitrogen) in a 2 ml tube for 1 hour. Further tissue disruption and homogenization was performed in three 1-min cycles with grinding beads using a TissueLyser II (Qiagen). RNA was purified from the homogenized sample with Direct-Zol RNA minipreps (Zymo, Irvine, USA), following the manufacturer's protocol. Quantity and quality were assessed using a RNA Pico chip on a Bioanalyzer 2100 (Agilent Technologies, Santa Clara, USA).

High-throughput 16S rRNA gene amplicon sequencing and processing

In order to determine the taxonomic complexity of the hologenome, high-throughput 16S rRNA gene amplicon sequencing was performed as described by Maus et al. [46]. Primers Pro341F (5′-CCTACGGGGNBGCASCAG-3′) and Pro805R (5′-GACTACNVGGGTATCTAATCC-3′) [47] were used to amplify the hypervariable regions V3 and V4 of the 16S rRNA gene in Bacteria and Archaea as well as in single-celled algae and other eukaryotes. Multiplex identifier (MID) tags and Illumina-specific sequencing adaptors were used for two PCR steps. The PCR products, featuring a length of 460 bp, were purified using AMPureXP magnetic beads (Beckman Coulter, Brea, USA). Quality and quantity assessments of 16S rRNA gene amplicons were done using the Agilent 2100 Bioanalyzer system. Then, amplicons were pooled in equimolar amounts for subsequent Illumina MiSeq sequencing (Illumina, San Diego, USA), applying the paired-end protocol. Adapter and primer trimming were performed through an *in-house* pipeline [48]. For amplicon processing, a pipeline including FLASH v.1.2.11 [49], USEARCH v.8.1 [50], UPARSE v.10.0.240 [51], and the RDP classifier v.2.9 [52] was used as described recently [46,53,54]. All sequences that were not merged by FLASH using default settings were filtered out. In addition, sequences with > 1 N (ambiguous base) in the sequence read and expected errors > 0.5 were also discarded. Resulting data were processed, operational taxonomic units (OTUs) clustered using USEARCH, and taxonomically classified with the RDP classifier in 16S modus. Only hits featuring a confidence value > 0.8 were considered. Finally, obtained raw sequence reads were mapped back onto the OTU sequences in order to get quantitative assignments.

Whole hologenome sequencing, binning, assembly, and annotation of the *P. rus* genome

To obtain hologenome sequence data, a whole-genome-shotgun PCR-free (TruSeq PCR-free DNA Sample Prep Kit; Illumina) and a 10 kbp mate-pair libraries (Nextera Mate Pair Sample Preparation Kit; Illumina) were generated based on the manufacturer’s protocols. Both libraries were sequenced

in two HiSeq 1500 rapid runs in paired-end mode (2 x 250 bp). After sequencing and processing of the raw data with Trimmomatic v.0.32 [55] and an *in-house* pipeline based on CASAVA v.1.8.2. (Illumina) [48], a *de novo* metagenome assembly was performed using the Ray Meta assembler v.2.3.0 [56] with a *k*-mer size of 41 and default settings. All processed raw reads were aligned to the assembled metagenome contigs using Bowtie v.2.2.4 [57]. The resulting bam file was sorted and read mapping statistics were calculated by applying bamtools v.2.4.1 [58]. To sort the metagenome contigs into genome bins, MetaBAT v.0.21.3 [38] was used by applying default settings. Here, it combines the formation of the tetra-nucleotide frequency and the contig abundance probabilities in order to produce high-quality genome bins out of a large hologenomic dataset. Coral raw reads were extracted by means of read mapping to all coral bins and later reassembled using the gsAssembler v.2.8 (Roche Diagnostics, Mannheim, Germany), applying the heterozygote mode with default settings. Gene prediction in the reconstructed *P. rus* draft genome was done with GeneMark v.4.3.2. [59]. The functional annotation of predicted genes was conducted within the GenDBE annotation platform for eukaryotes [60]. To assess the quality of gene prediction, RNAseq data were mapped onto the genome sequences. Results were compared manually and by bioinformatics approaches (e.g., bedtools [61]) to the gene prediction results. Due to the lack of high quality functional annotations of coral genes in public databases, a homology based assignment of gene functions is not suitable. Therefore only an annotation describing the structure of the genes based on functional domains was performed with InterProScan [62].

Sequencing of cDNA libraries

In total, 2.25 µg of RNA were used for library preparation with the TruSeq Stranded mRNA Sample Preparation Kit (Illumina). Sequencing of the cDNA library was carried out on the Illumina HiSeq 1500 platform following a modified protocol of Verwaaijen et al. [63]. The sample was paired-end sequenced in a rapid run with 2 × 75 bp cycles. Base calling and data processing was accomplished

using *in-house* software (see above). Obtained reads were quality filtered (> Q30) by applying the FASTX tool kit [64]. The data set was mapped onto the established *P. rus* genome assembly using tophat v.2.1.1 [65]. Two mismatches were allowed to account for possible sequencing errors and allelic variants of the diploid *P. rus* genome. The sequence read analysis platform ReadXplorer v.2.2.3. [66,67] was used for visualization and further analysis of the data. Reads per kilobase per million reads (RPKM) values were calculated from exported read count tables, using the *single best match* option for each library. In addition, gene prediction results were confirmed with ReadXplorer. The RNAseq and the draft genome data are available on the EBI Annotare server (Project-ID: E-MTAB-6535) and in European Nucleotide Archive (ENA) (Accession numbers: OKRP01000001-OKRP01014982), respectively.

_K_-mer-based genomic features estimation and assembly quality assessment

The size of the *P. rus* genome was estimated through short-sequence substring (*k*-mers) frequencies of the reads used for the assembly. Different *k*-mer sizes were implemented, ranging from 17 to 25 bp. Their *k*-mer count distributions were calculated with Jellyfish v.2.0 [68]. In order to account for the lack of cytometric measures for *P. rus* [69] and the highly variable estimations reported for cnidarians [22,41], these profile distributions were used to perform three independent genome size estimations (see supplementary methods for detailed information about genome size estimations). The first approach corresponded to the Waterman's estimation method [70], which is based on the relationship between the number of used bases and *k*-mers obtained. The second approach implemented the heterozygous mode of the gce program v.1.0 [39], that, in addition to Waterman's estimation, takes sequencing errors and coverage bias in the *k*-mer distribution into account. The third approach utilized the GenomeScope web interface tool v.1.0. [35], which uses a negative binomial mixture model to account for the genomic complexity of *k*-mer frequency profiles. In addition, the latter approach measures the relative abundances of heterozygous, homozygous,

unique, and two-copy sequences. The performance of these three approaches was evaluated based on paired-end reads from the two genomic datasets for which flow cytometry estimations are available (i.e., *A. digitifera* [16] with an estimated haploid size of ~420 Mbp, and *E. pallida* [22] with ~260 Mbp). As these analyses indicated that there is no single superior methodology for coral genome size estimations, we calculated the median genome size based on the 21-mer distribution in the three approaches conducted (see supplementary methods for detailed description).

Standard statistics for assessing the assembly's contiguity were calculated using QUAST v. 4.4 [71] and the *assemblathon_stats.pl* script [36]. Completeness was estimated by i) calculating the content of metazoan BUSCOs (e-value: 0.001, dataset v.3.0.2) [72] in the genome and the gene datasets, and ii) determining the presence of orthologs previously described within a comparative genomic framework of reef cnidarians at various taxonomic levels (Reef Genomics database [19]) using *blastx* (e-value: 0.001, blast v.2.2.30 [73]). Finally, the usefulness was estimated by the percentage of genome size in scaffolds higher or equal to the average gene size of invertebrate model organisms, following Bradnam et al. [36] and using the latest protein-coding annotations of the Ensembl 89 Genes dataset [74] for *Caenorhabditis elegans* (WBcel235), *Ciona intestinalis* (KH), *Ciona savignyi* (CSAV 2.0), *Drosophila melanogaster* (BDGP6), and *Saccharomyces cerevisiae* (R64-1-1).

Comparative quality assessment of binning- and symbiont-free-based cnidarian assemblies

In order to assess the quality of our hologenome binning-based assembly, we compared it to literature data from the symbiont-free-based genome assemblies of *N. vectensis* v.1.0 [21], *A. digitifera* v.1.1 [16,75] and *E. pallida* v.1.1 [22] (Tables S1 and S2). The comparison was based on the operational criteria completeness with six parameters, contiguity with seven parameters, and usefulness with two parameters (see Table S3 for parameter details). Modified z-scores (*sensu* Bradnam et al. [36]) were calculated individually for the respective parameters (Table S3). Then the

individual scores were summed up and the standard deviation from the median value was used to assign the quality levels good (+), average (O) and poor (-) to each assembly.

Availability of supporting data and materials

New sequence data used in this manuscript has been submitted to the European Bioinformatics Institute (EMBL-EBI) database under the project IDs E-MTAB-6535, PRJEB23570, PRJEB25185, and the accession numbers OKRP01000001-OKRP01014982.

Additional Files

Figure S1: Krona chart depicting the 16S rRNA gene amplicon taxonomical profile.

Figure S2: Genome size estimation for *Porites rus* using *k*-mer based approaches.

Table S1: *Porites rus*, *Acropora digitifera*, *Nematostella vectensis,* and *Exaiptasia pallida* genome accession numbers.

Table S2: Summary genome assemblies' statistics.

Table S3: Summary operational criteria completeness, contiguity, and usefulness.

Table S4: *Porites rus k*-mer statistics.

Table S5: Summary *k*-mer based genome size estimation.

Supplemental methods: Detailed description of the *k*-mer based genome size estimation.

Declarations

List of abbreviations

16S rRNA: 16S ribosomal RNA; BLAST: Basic local alignment search tool; BUSCOs: Benchmarking universal single-copy orthologs; cDNA: Complementary DNA; EC numbers: Enzyme commission

number; EMBL: European Molecular Biology Laboratory; ENA: European Nucleotide Archive; gDNA: Genomic DNA; GO: Gene ontology; MID: Multiplex identifier; NCBI: National Center for Biotechnology Information; OTUs: Operational taxonomic units; PCR: Polymerase chain reaction; RPKM: Reads per kilobase per million reads.

Statement on ethics approval and consent

Not applicable

Consent for Publication

Not applicable

Competing interests

The authors declare that they have no competing interests.

Funding

The German Academic Exchange Service (DAAD, Grant number 91559856) and the CEMarin (Grant number: CO8JUCE) provided financial support to JSC for this research. The bioinformatics support of the BMBF-funded project 'Bielefeld-Giessen Center for Microbial Bioinformatics − BiGi' (Grant number 031A533) within the German Network for Bioinformatics Infrastructure (de.NBI) is acknowledged.

Authors' contributions

JSC, DW, CRP, JK and TW initiated the study, designed the experiments, and reviewed the data. JSC, DW, CRP, AS and AW designed and performed the experiments. JSC, DW, CRP, OR and AS analyzed the data. TW and JK provided funding and laboratory resources. TW supervised the project. JSC

wrote the original draft. JSC, DW, CRP, AS, OR, JK and TW contributed to the writing and reviewed the manuscript.

Acknowledgements

This study is part of the global change simulation project 'Ocean 2100' at Justus Liebig University Giessen, initiated by the Colombian-German Center of Excellence in Marine Sciences (CEMarin). The authors would like to thank Patrick Schubert and Jessica Reichert for their support, and for providing the *Porites rus* materials from the aquaculture facilities at Justus Liebig University Giessen. We thank Silvia Nachtigall and Tobias Warmann (Justus Liebig University Giessen) for laboratory assistance. The valuable discussions with Maren Ziegler (King Abdullah University of Science and Technology KAUST, Saudi Arabia) are also gratefully acknowledged. We would also like to thank Jochen Blom (Bioinformatics and Systems Biology, Justus Liebig University Giessen) for his help in an early stage of the project.

References

1. Bordenstein SR, Theis KR. Host biology in light of the microbiome: Ten principles of holobionts and hologenomes. PLoS Biol. 2015;13:e1002226.

2. Nelson KE, Weinstock GM, Highlander SK, Worley KC, Creasy HH, Wortman JR, et al. A catalog of reference genomes from the human microbiome. Science. 2010;328:994–9.

3. Hongoh Y. Diversity and genomes of uncultured microbial symbionts in the termite gut. Biosci Biotechnol Biochem. 2010;74:1145–51.

4. Kuo A, Kohler A, Martin FM, Grigoriev IV. Expanding genomics of mycorrhizal symbiosis. Front Microbiol. 2014;5:582.

5. Margulis L, Fester R. Eds. Symbiosis as a source of evolutionary innovation. Speciation and morphogenesis. Cambridge: The MIT Press; 1991.

6. Rosenberg E, Zilber-Rosenberg I. The hologenome concept: human, animal and plant microbiota. Heidelberg. Springer; 2013.

7. Rohwer F, Seguritan V, Azam F, Knowlton N. Diversity and distribution of coral-associated bacteria. Mar Ecol Prog Ser. 2002;243:1–10.

8. Muscatine L, Porter JW. Reef Corals : mutualistic symbioses adapted to nutrient-poor environments. Bioscience. 1977;27:454–60.

9. Wegley L, Edwards R, Rodriguez-Brito B, Liu H, Rohwer F. Metagenomic analysis of the microbial community associated with the coral *Porites astreoides*. Environ Microbiol. 2007;9:2707–19.

10. del Campo J, Pombert J-F, Šlapeta J, Larkum A, Keeling PJ. The “other” coral symbiont: *Ostreobium* diversity and distribution. ISME. 2017;11:296–9.

11. Le Campion-Alsumard T, Golubic S, Priess K. Fungi in corals: Symbiosis or disease? Interaction between polyps and fungi causes pearl-like skeleton biomineralization. Mar Ecol Prog Ser. 1995;117:137–48.

12. Blackall LL, Wilson B, van Oppen MJH. Coral - The world’s most diverse symbiotic ecosystem. Mol Ecol. 2015;24:5330–47.

13. Weynberg KD, Wood-Charlson EM, Suttle CA, van Oppen MJH. Generating viral metagenomes from the coral holobiont. Front Microbiol. 2014;5:1–11.

14. Wood-Charlson EM, Weynberg KD, Suttle CA, Roux S, van Oppen MJH. Metagenomic characterization of viral communities in corals: mining biological signal from methodological noise. Environ Microbiol. 2015;17:3440–49.

15. Mies M, Sumida PYG, Rädecker N, Voolstra CR. Marine invertebrate larvae associated with Symbiodinium: a mutualism from the start?. Front Ecol Evol. 2017;5:1–11.

16. Shinzato C, Shoguchi E, Kawashima T, Hamada M, Hisata K, Tanaka M, et al. Using the *Acropora digitifera* genome to understand coral responses to environmental change. Nature. 2011;476:320–3.

17. Shoguchi E, Shinzato C, Kawashima T, Gyoja F, Mungpakdee S, Koyanagi R, et al. Draft assembly of the *Symbiodinium minutum* nuclear genome reveals dinoflagellate gene structure. Curr Biol. 2013;23:1399–408.

18. Shinzato C, Inoue M, Kusakabe M. A snapshot of a coral “holobiont”: a transcriptome assembly of the scleractinian coral, *Porites*, captures a wide variety of genes from both the host and symbiotic zooxanthellae. PLOS ONE. 2014;9:e85182.

19. Bhattacharya D, Agrawal S, Aranda M, Baumgarten S, Belcaid M, Drake JL, et al. Comparative genomics explains the evolutionary success of reef-forming corals. elife. 2016;5: e13288.

20. Kajitani R, Toshimoto K, Noguchi H, Toyoda A, Ogura Y, Okuno M, et al. Efficient *de novo* assembly of highly heterozygous genomes from whole-genome shotgun short reads. Genome Res. 2014;24:1384–95.

21. Putnam NH, Srivastava M, Hellsten U, Dirks B, Chapman J, Salamov A, et al. Sea anemone genome reveals ancestral eumetazoan gene repertoire and genomic organization. Science. 2007;317:86–94.

22. Baumgarten S, Simakov O, Esherick LY, Liew YJ, Lehnert EM, Michell CT, et al. The genome of *Aiptasia*, a sea anemone model for coral symbiosis. Proc Natl Acad Sci USA. 2015;112:11893–98.

23. Wang X, Liew YJ, Li Y, Zoccola D, Tambutte S, Aranda M. Draft genomes of the corallimorpharians *Amplexidiscus fenestrafer* and *Discosoma* sp. Mol Ecol Resour. 2017;1–9.

24. Murray-Roberts J, Wheeler AJ, Freiwald A, Cairns SD. Cold-water corals: the biology and geology of deep-sea coral habitats. Cambridge: Cambridge University Press; 2010.

25. Bongaerts P, Riginos C, Brunner R, Englebert N, Smith SR. Deep reefs are not universal refuges: reseeding potential varies among coral species. Sci Adv. 2017;3:e160237.

26. Ghurye JS, Pop M. Metagenomic assembly: overview, challenges and applications. Yale J Biol Med. 2016;89:353–62.

27. Sharpton TJ. An introduction to the analysis of shotgun metagenomic data. Front Plant Sci. 2014;5:1–14.

28. Krause L, Diaz NN, Goesmann A, Kelley S, Nattkemper TW, Rohwer F, et al. Phylogenetic classification of short environmental DNA fragments. Nucleic Acids Res. 2008;36:2230–9.

29. Albertsen M, Hugenholtz P, Skarshewski A, Nielsen KL, Tyson GW, Nielsen PH. Genome sequences of rare, uncultured bacteria obtained by differential coverage binning of multiple metagenomes. Nat Biotechnol. 2013;31:533–8.

30. Mrázek J. Phylogenetic signals in DNA composition: Limitations and prospects. Mol Biol Evol. 2009;26:1163–9.

31. Saeed I, Tang SL, Halgamuge SK. Unsupervised discovery of microbial population structure within metagenomes using nucleotide base composition. Nucleic Acids Res. 2012;40:e34.

32. Ji P, Zhang Y, Wang J, Zhao F. MetaSort untangles metagenome assembly by reducing microbial community complexity. Nat Commun. 2017;8:14306.

33. Neave MJ, Michell CT, Apprill A, Voolstra CR. *Endozoicomonas* genomes reveals functional adaptation and plasticity in bacterial strains symbiotically associated with diverse marine hosts. Sci Rep. 2017;7:40579.

34. Diehl E, Jauker B, Albrecht C, Wilke T. Gießen: University Collections: Justus Liebig University Gießen. In: Beck LA, editor. Zoological Collections of Germany: The animal kingdom and its amazing plenty at museums and Universities. Cham: Springer; 2018. p. 373–81.

35. Takahashi S, Tomita J, Nishioka K, Hisada T, Nishijima M. Development of a prokaryotic universal primer for simultaneous analysis of Bacteria and Archaea using next-generation sequencing. PLOS One. 2014;9:e105592.

36. Kang DD, Froula J, Egan R, Wang Z. MetaBAT, an efficient tool for accurately reconstructing single genomes from complex microbial communities. PeerJ. 2015;3:e1165.

37. Lomsadze A, Burns PD, Borodovsky M. Integration of mapped RNA-Seq reads into automatic training of eukaryotic gene finding algorithm. Nucleic Acids Res. 2014;42:e119.

38. Vurture GW, Sedlazeck FJ, Nattestad M, Underwood CJ, Fang H, Gurtowski J, et al. GenomeScope: fast reference-free genome profiling from short reads. Bioinformatics. 2017; 33:2202-2204.

39. Bradnam KR, Fass JN, Alexandrov A, Baranay P, Bechner M, Birol I, et al. Assemblathon 2: evaluating *de novo* methods of genome assembly in three vertebrate species. GigaScience. 2013; 2:10.

40. Wrighton KC, Thomas BC, Sharon I, Miller CS, Castelle CJ, VerBerkmoes NC, et al. Fermentation, hydrogen, and sulfur metabolism in multiple uncultivated bacterial phyla. Science. 2012;337:1661–5.

41. Liu B, Shi Y, Yuan J, Hu X, Zhang H, Li N, et al. Estimation of genomic characteristics by analyzing *k*-mer frequency in *de novo* genome projects. arXiv. 2013;1308.2012.

42. Li Z, Chen Y, Mu D, Yuan J, Shi Y, Zhang H, et al. Comparison of the two major classes of assembly algorithms: overlap-layout-consensus and de-bruijn-graph. Brief Funct Genomics. 2012;11:25–37

43. Adachi K, Miyake H, Kuramochi T, Mizusawa K, Okumura S. Genome size distribution in phylum Cnidaria. Fish Sci. 2017;83:107–12.

44. Sims D, Sudbery I, Ilott NE, Heger A, Ponting CP. Sequencing depth and coverage: key considerations in genomic analyses. Nat Rev Genet. 2014;2:121–32.

45. Nagarajan N, Pop M. Sequence assembly demystified. Nat Rev Genet. 2013;14:157–67.

46. Banaszak AT. Optimization of DNA extraction from a scleractinian coral for the detection of thymine dimers by immunoassay. Photochem Photobiol. 2007;83:833–8.

47. Maus I, Kim YS, Wibberg D, Stolze Y, Off S, Antonczyk S, et al. Biphasic study to characterize agricultural biogas plants by high-throughput 16S rRNA gene amplicon sequencing and microscopic analysis. J Microbiol Biotechnol. 2016;27:321–34.

48. Wibberg D, Andersson L, Rupp O, Goesmann A, Pühler A, Varrelmann M, et al. Genome analysis of the sugar beet pathogen *Rhizoctonia solani* AG2-2IIIB revealed high numbers in secreted proteins and cell wall degrading enzymes. BMC Genomics. 2016;17:245.

49. Magoč T, Salzberg SL. FLASH: fast length adjustment of short reads to improve genome assemblies. Bioinformatics. 2011;27:2957–63.

50. Edgar RC. Search and clustering orders of magnitude faster than BLAST. Bioinformatics. 2010;26:2460–1.

51. Edgar RC. UPARSE: highly accurate OTU sequences from microbial amplicon reads. Nat Methods. 2013;10:996–8.

52. Wang Q, Garrity GM, Tiedje JM, Cole JR. Naïve Bayesian classifier for rapid assignment of rRNA sequences into the new bacterial taxonomy. Appl Environ Microbiol. 2007;73:5261–7.

53. Liebe S, Wibberg D, Winkler A, Pühler A, Schülter A, Varrelmann M. Taxonomic analysis of the microbial community in stored sugar beets using high-throughput sequencing of different marker genes. FEMS Microbiol Ecol. 2016;92:1–12.

54. Theuerl S, Kohrs F, Benndorf D, Maus I, Wibberg D, Schülter A, et al. Community shifts in a well-operating agricultural biogas plant: how process variations are handled by the microbiome. Appl Microbiol Biotechnol. 2015;99:7791–803.

55. Bolger AM, Lohse M, Usadel B. Trimmomatic: a flexible trimmer for Illumina sequence data. Bioinformatics. 2014;30:2114–20.

56. Boisvert S, Raymond F, Godazaridis E, Laviolette E, Corbeil J. Ray Meta: scalable *de novo* metagenome assembly and profiling. Genome Biology. 2012;13:R122.

57. Langmead B, Salzberg SL. Fast gapped-read alignment with Bowtie 2. Nat Methods. 2012;9:357–9.

58. Barnett DW, Garrison EK, Quinlan AR, Strömberg MP, Marth GT. Bamtools: A C++ API and toolkit for analyzing and managing BAM files. Bioinformatics. 2011;27:1691–2.

59. Rupp O, Becker J, Brinkrolf K, Timmermann C, Borth N, Pühler A, et al. Construction of a public CHO cell line transcript database using versatile bioinformatics analysis pipelines. PLOS ONE. 2014;9:e85568.

60. Quinlan AR, Hall IM. BEDTools: A flexible suite of utilities for comparing genomic features. Bioinformatics. 2010;26:841–2.

61. Jones P, Binns D, Chang HY, Fraser M, Li W, McAnulla C, et al. InterProScan 5: Genome-scale protein function classification. Bioinformatics. 2014;30:1236–40.

62. Verwaaijen B, Wibberg D, Kröber M, Winkler A, Zrenner R, Bednarz H, et al. The *Rhizoctonia solani* AG1-IB (isolate 7/3/14) transcriptome during interaction with the host plant lettuce (*Lactuca sativa* L.). PLOS ONE. 2017;12:e0177278.

63. HannonLab. FASTX toolkit. https://hannonlab.cshl.edu/fastx_toolkit/. Accessed 15 Oct 2017.

64. Kim D, Pertea G, Trapnell C, Pimentel H, Kelley R, Salzberg SL. TopHat2: accurate alignment of transcriptomes in the presence of insertions, deletions and gene fusions. Genome Biology. 2013;14:R36.

65. Hilker R, Stadermann KB, Doppmeier D, Kalinowski J, Stoye J, Straube J, et al. ReadXplorer - Visualization and analysis of mapped sequences. Bioinformatics. 2014;30:2247–54.

66. Hilker R, Bernd Stadermann K, Schwengers O, Anisiforov E, Jaenicke S, Weisshaar B, et al. ReadXplorer 2 - detailed read mapping analysis and visualization from one single source. Bioinformatics. 2016;32:3702–8.

67. Marçais G, Kingsford C. A fast, lock-free approach for efficient parallel counting of occurrences of *k*-mers. Bioinformatics. 2011;27:764–70.

68. Gregory TR, Nicol JA, Tamm H, Kullman B, Kullman K, Leitch IJ, et al. Eukaryotic genome size databases. Nucleic Acids Res. 2007;35:332-8.

69. Li X, Waterman MS. Estimating the repeat structure and length of DNA sequences using l-Tuples. Genome Res. 2003;13:1916–22.

70. Gurevich A, Saveliev V, Vyahhi N, Tesler G. QUAST: quality assessment tool for genome assemblies. Bioinformatics. 2013;29:1072–5.

71. Simão FA, Waterhouse RM, Ioannidis P, Kriventseva E V., Zdobnov EM. BUSCO: Assessing genome assembly and annotation completeness with single-copy orthologs. Bioinformatics. 2015;31:3210–2.

72. Altschul SF, Gish W, Miller W, Myers EW, Lipman DJ. Altschul et al. Basic local alignment search tool. J Mol Biol. 1990;215:403–10.

73. Yates A, Akanni W, Ridwan Amode M, Barrel D, Billis K, Carvalho-Silva D, et al. Ensembl 2016. Nucleic Acids Res. 2016;44:710–6.

74. Shinzato C, Mungpakdee S, Arakaki N, Satoh N. Genome-wide SNP analysis explains coral diversity and recovery in the Ryukyu Archipelago. Sci Rep. 2015;5:1–8.

Figure 1

Click here to download Figure Celis_Fig1.pdf

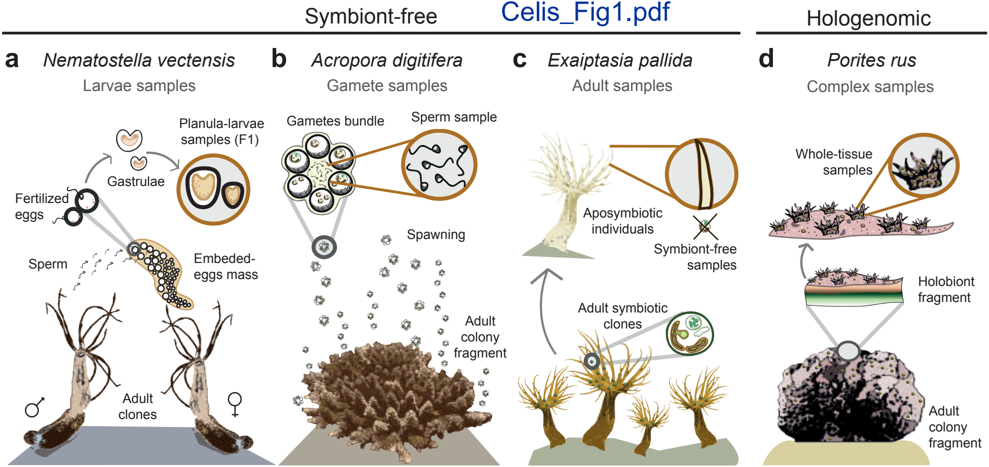

Figure 2

Click here to download Figure Celis_Fig2.pdf

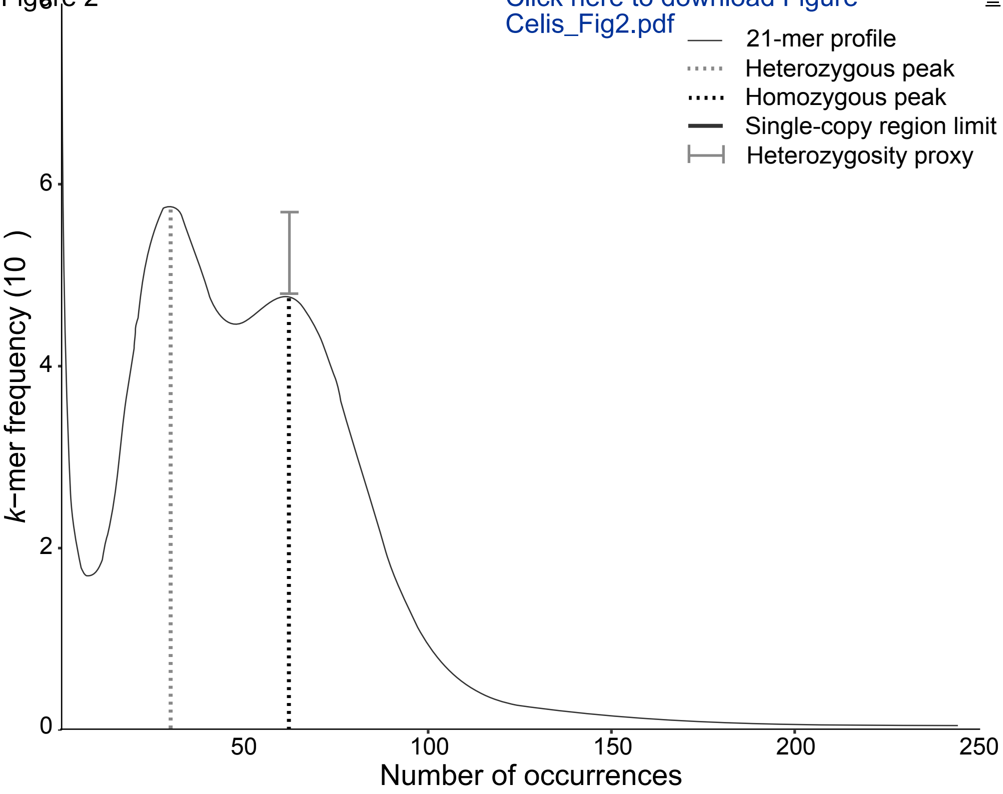

Figure 3

Click here to download Figure Celis_Fig3.pdf

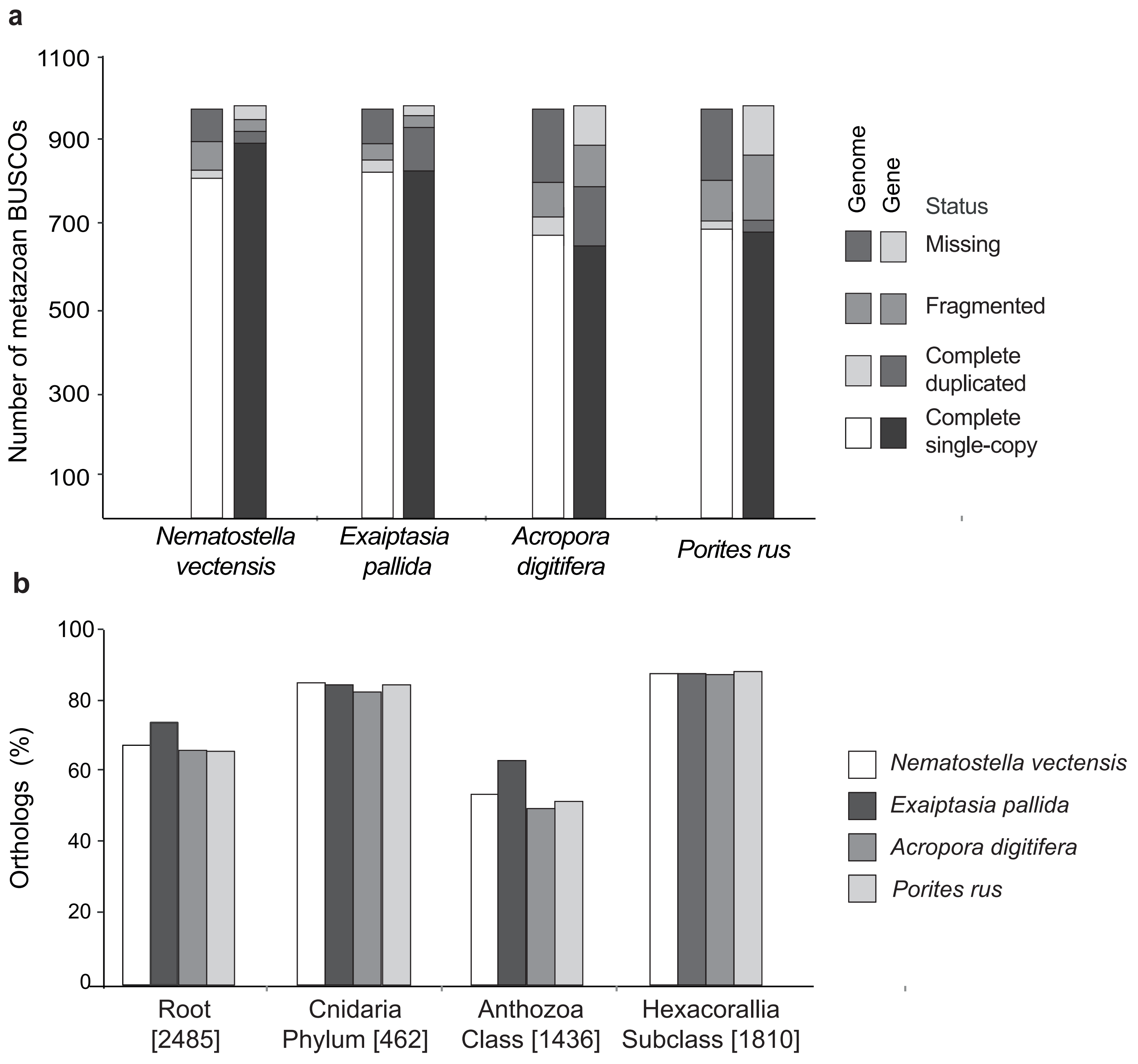

Figure 4

Click here to download Figure Celis_Fig4.pdf

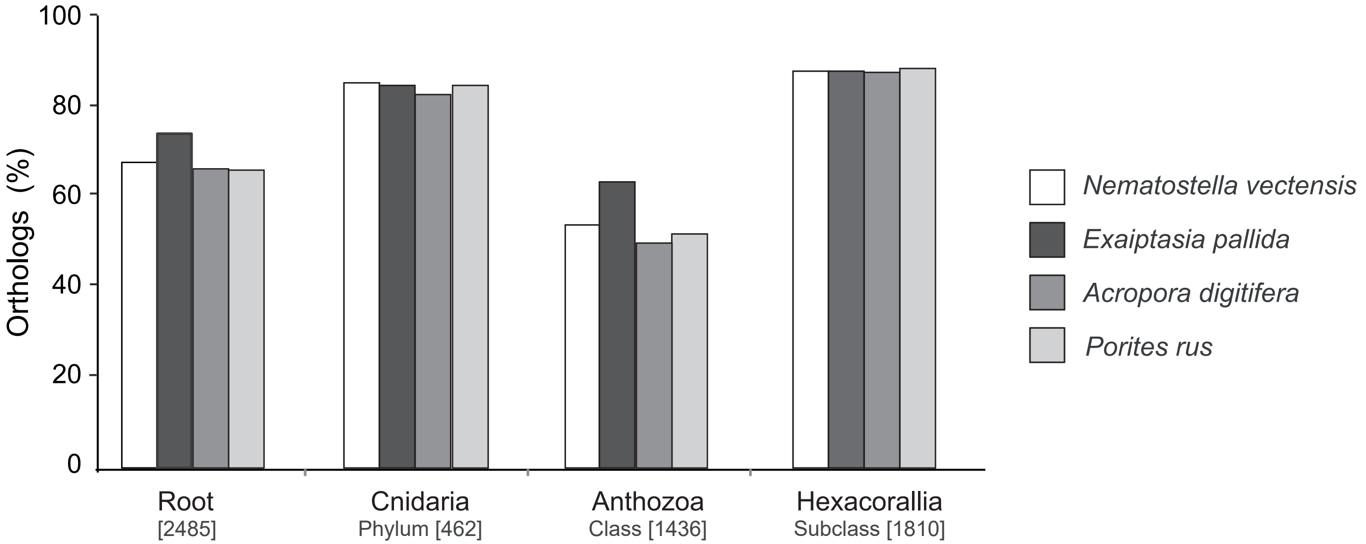

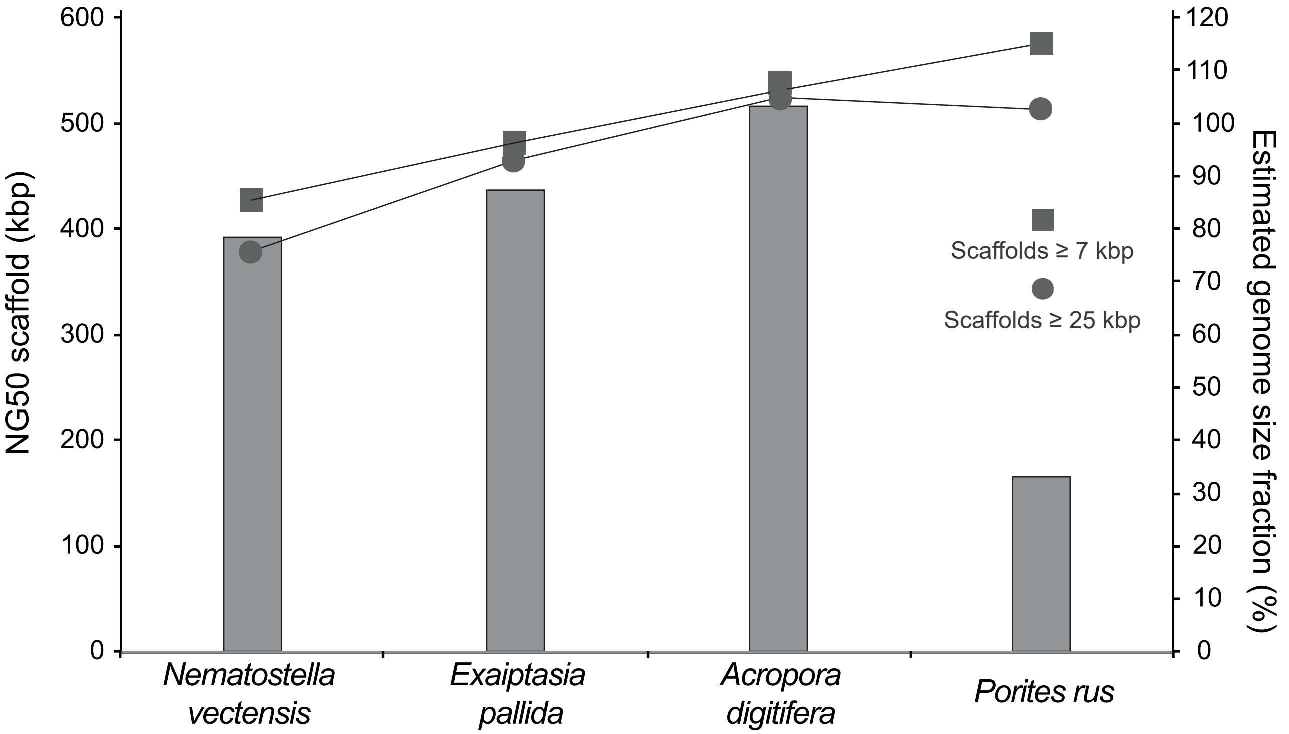

600
500
400
300
200
100
0
NG50 scaffold (kbp)
120
110
100
90
80
70
60
50
40
30
20
10
0
Estimated genome size fraction (%)
Scaffolds ≥ 7 kbp
Scaffolds ≥ 25 kbp
Nematostella vectensis
Exaiptasia pallida
Acropora digitifera
Porites rus

Figure 6

Click here to download Figure Celis_Fig6.pdf

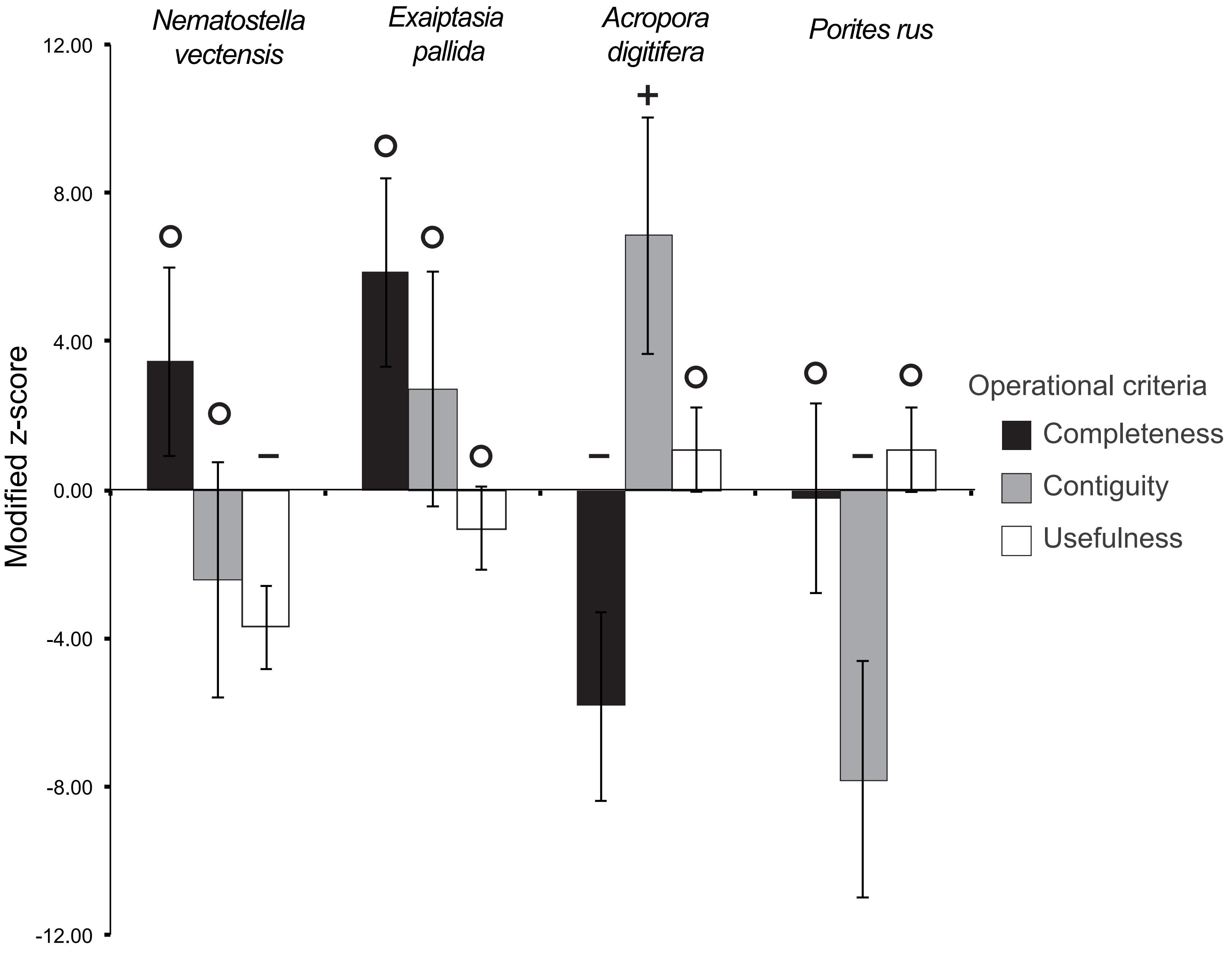

Supplementary Figure 1

Click here to access/download
Supplementary Material
Supplemental_Fig_S1.pdf

Supplementary Figure 2

Click here to access/download
Supplementary Material
Supplemental_Fig_S2.pdf

Supplementary Table 1

Click here to access/download
Supplementary Material
Supplemental_Table_S1 .xls

Supplementary Table 2

Click here to access/download
Supplementary Material
Supplemental_Table_S2 .xls

Supplementary Table 3

Click here to access/download
Supplementary Material
Supplemental_Table_S3.xls

Supplementary Table 4

Click here to access/download
Supplementary Material
Supplemental_Table_S4.xls

Supplementary Table 5

Click here to access/download
Supplementary Material
Supplemental_Table_S5.xls

Click here to access/download
Supplementary Material
Celis et al, 2018_Supplemental_Methods.doc

 Click here to download Personal Cover Celis et al, 2018_Cover letter_GigaScience.pdf

JUSTUS-LIEBIG-
UNIVERSITÄT
GIESSEN

Cover Letter Submission *GigaScience*

February 26th, 2018

Dear Dr. Goodman,
Editor-in-Chief *GigaScience*

Please find attached our manuscript entitled: '**Binning Enables Efficient Host Genome Reconstruction in Cnidarian Holobionts**' for publication in *GigaScience*. Our study represents an important assessment of binning for facilitating host genome reconstruction of taxonomically complex holobionts.

Many cnidarians, including stony corals, form essential symbiotic associations with protists and bacteria. The taxonomical complexity of these 'holobionts' makes sequencing and assembling of coral genomes extremely challenging. In fact, previous genome sequencing projects of holobionts were based on 'symbiont-free' host DNA, i.e. an *in situ* reduction of taxonomic complexity. In this study, we evaluated the performance of binning as an *in silico* alternative for reconstructing the host genome of a model holobiont (*Porites rus*; Scleractinia). Our results demonstrate that binning may handle hologenomic samples well, providing enough binned reads for assembling the draft genome of the stony coral *P. rus* (EMBL-EBI ArrayExpress accession number: E-MTAB-6535). A comparative assessment of our operational criteria across cnidarian genomes revealed that the binning approach displayed comparable results to symbiont-free approaches in terms of completeness and usefulness, despite a high degree of fragmentation. In addition, we demonstrated that binning might supply data for exploratory *k*-mer based estimations of genomic features like genome size, heterozygosity and repetitive content. Thus, binning constitutes a powerful tool for an *in silico* reduction of the taxonomic complexity of holobiontic samples.

This study might be of general interest for geneticists and bioinformaticians as it provides an important milestone in taking binning from the metagenomic to the hologenomic and from the prokaryotic to the eukaryotic level. We therefore believe that our work could constitute a significant contribution to *GigaScience*.

We confirm that all authors approved the manuscript for submission and that the content of the manuscript has not been published, or submitted for publication elsewhere. All the authors declare no competing interests.

Sincerely yours,

Juan Sebastián Celis on behalf of all co-authors
