# Supplementary figures and images for: Binning enables efficient host genome reconstruction in cnidarian holobionts
Source: Gigascience. 2018 Jul 18;7(7):giy075. doi: 10.1093/gigascience/giy075 (PMC6049006; doi:10.1093/gigascience/giy075)

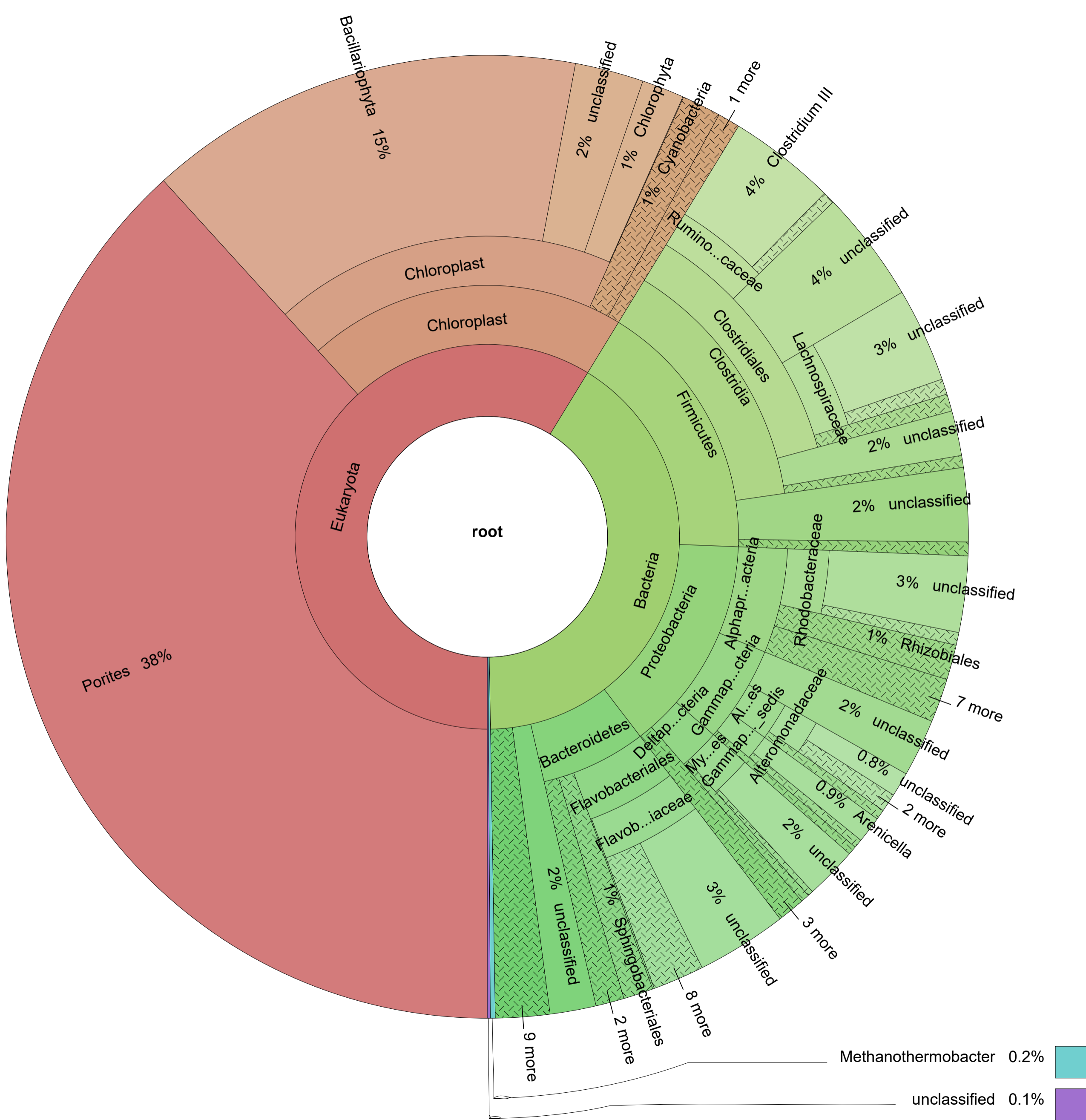

Supplement: Additional Files [file giy075_supplement_files.zip › Supplemental_Fig_S1.pdf]

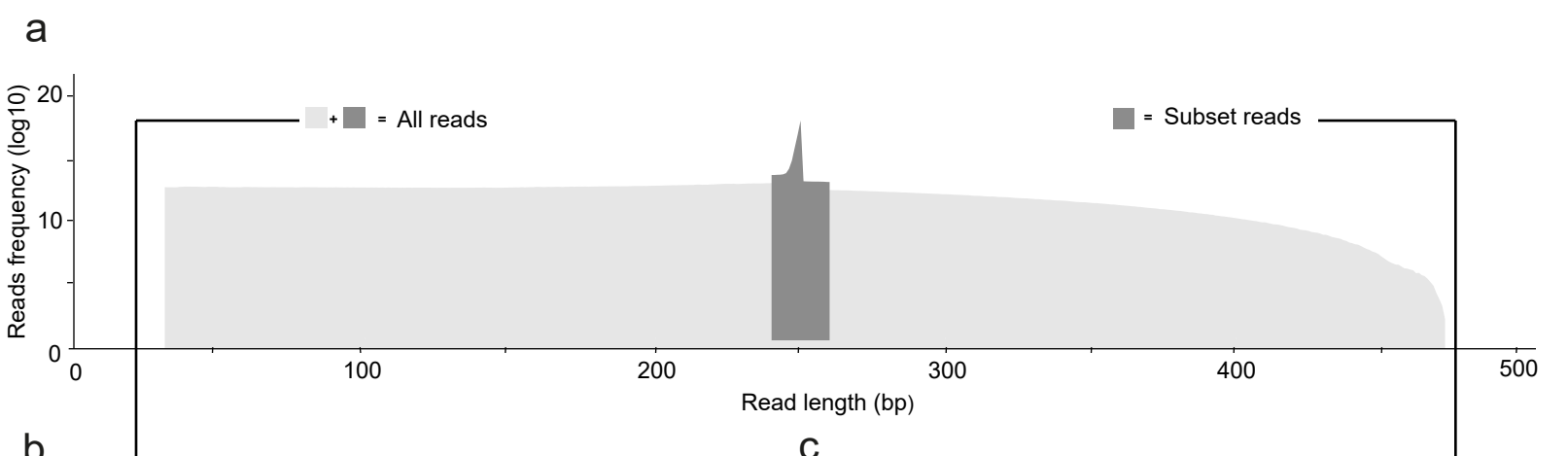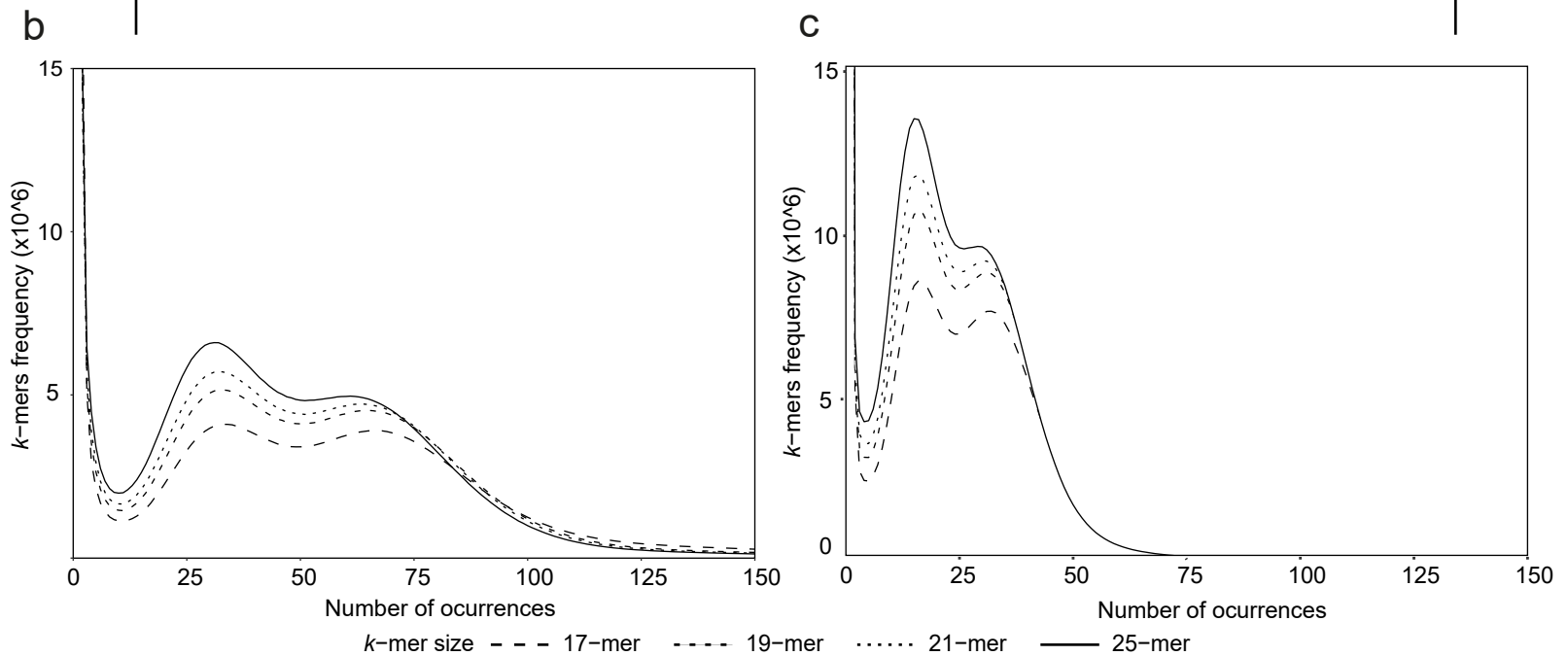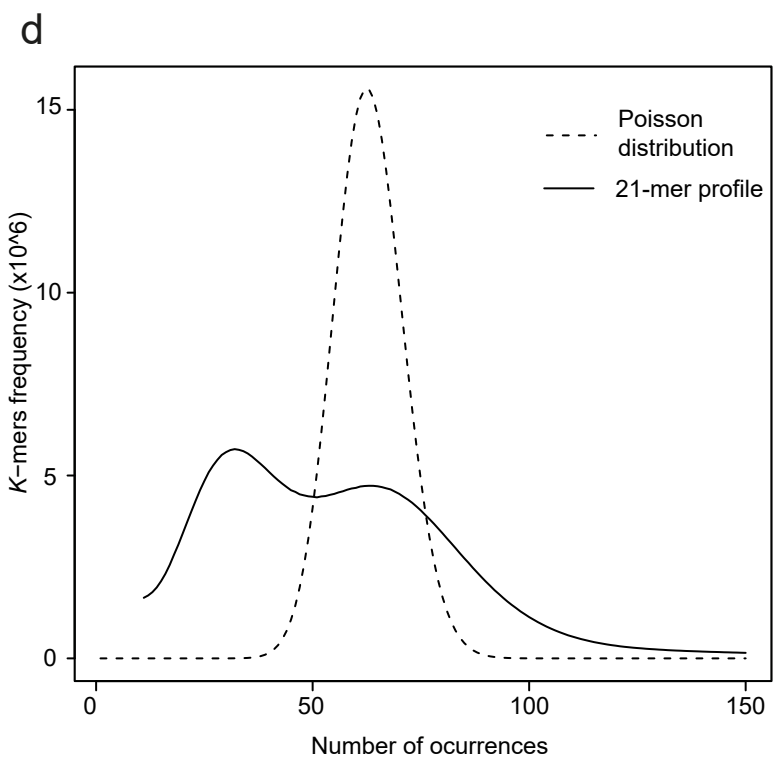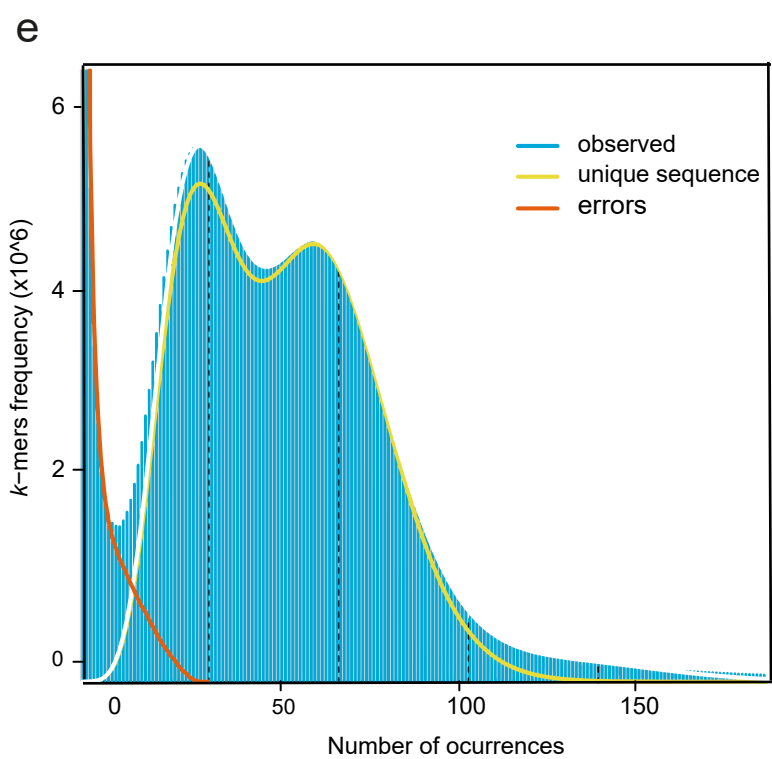

Supplement: Additional Files [file giy075_supplement_files.zip › Supplemental_Fig_S2.pdf]
