# Supplementary material for: Binning enables efficient host genome reconstruction in cnidarian holobionts
Source: Gigascience. 2018 Jul 18;7(7):giy075. doi: 10.1093/gigascience/giy075 (PMC6049006; doi:10.1093/gigascience/giy075)
Supplement: Response_to_Reviewer_Comments_Original_Submission.pdf [file giy075_response_to_reviewer_comments_original_submission.pdf]

Author's Response To Reviewer Comments

Close

We very much appreciate the time and effort of the two reviewers and the editor in assessing our paper, and we are grateful for their useful comments.

Below we list all critical comments and suggestions of the reviewers (C), together with our point-by-point reply (R).

Reviewer # 1 (Beate Slaby)

C1: I know that you did not aim at comparing different assembly methods. Yet, looking at the low contiguity of the assembly: did you test other assemblers? A small comparison would have been nice. But at this point, I don't think it is necessary to go back and do this, but rather include a clear statement that the results could vary/improve depending on the algorithms that perform best with the data at hand

R: As pointed out by the reviewer, our intention was not to compare assembler performances but to test whether binning is efficient in reducing hologenomic data complexity, facilitating independent host genome assembly. In our study, the assembler was chosen based on the features of the binned dataset and our specific research question. To make this clearer to the reader, we included a statement on this issue in the revised manuscript in lines 330-337.

C2: Line 167: "Blastx" is in italics, "r2cat" is not

R: Corrected.

C3: Table 1: Why not include the values for the other genomes (Table S2) here? It would be good to have this comparison directly in the paper and not only in the supplements

R: Table S2 is included in the supplements rather than in the main manuscript text due to its size. For such large tables, GigaScience guidelines states that larger datasets or tables should be uploaded as additional files.

C4: Figures: there is a mix-up in the attached figures. "Fig.4" is actually again "Fig. 3b", "Fig. 5" should be "Fig. 4" and so on

R: The numbering was corrected.

Reviewer #2 (Thomas Hackl)

C1: I have not had any experience with gsAssembler, and I do not want to get in the "what is the best assembler" discussion, but I'd be curious as to why you chose gsAssembler. I would not have been my first choice (and similar for Ray Meta). My guess is that you could get better results with a combo: metaSPAdes + dipSPAdes.

R: This is a valid point. We decided to follow a conservative assembly strategy in order to reduce the probability of misassemblies. gsAssembler is not known for producing superior

N50 values. Rather it has been shown to produce low amounts of misassembles without the need for extensive setting optimization. Thus it is adequate when working with large and complex datasets, which also matches our own experiences with assembling genomes starting from high-complexity datasets. Finally, as clearly pointed out, the "best assembler" discussion is pointless, since the assembly quality is defined by the research question behind each genome assembly. Our manuscript was not intended to assess assemblers´ performance, but rather to show how binning can provide the data for downstream assembly. We included a brief statement about the assembly quality definition in lines 330-337.

C2: Have you thought about exploring reads not mapped, or mapping to unbinned contigs in combination with a gap-filling tool or similar to improve contiguity of the assembly. I could see a lot of the assembly gaps being related to reads not mapped to the contigs in your 13 host bins.

R: The use of gap-filling software works efficiently for small and low-complexity bacterial and fungal genomes, which generally contain small gaps. In contrast, eukaryotic genome assemblies display larger gaps within high repetitive genomic regions. Therefore, the application of gap-filling software in our coral assembly may result in artificially closed gaps. In addition, the use of unbinned contigs for the coral host genome assembly might lead to erroneous inclusion of symbiont contigs (Metagenomic contamination) in the host genome assembly.

C3: Binning is quite sensitive to contig length. I think that this approach could be even more powerful when used in combination with PacBio/Nanopore sequencing. You would get much more long contigs in the initial assembly, therefore better binning, and in turn higher completeness and, in particular, higher contiguity in the host-only assembly.

R: This is a good point. The performance of binning increases with the length of the assembled metagenomic contigs, leading to improved host genome assemblies, as suggested by the reviewer. Therefore, we included a brief comment (lines 322-324) about the advantages of including long read data (PacBio/Oxford Nanopore) for future binning-based host genome assemblies.

Close
