# Supplementary material for: Binning enables efficient host genome reconstruction in cnidarian holobionts
Source: Gigascience. 2018 Jul 18;7(7):giy075. doi: 10.1093/gigascience/giy075 (PMC6049006; doi:10.1093/gigascience/giy075)
Supplement: Reviewer_2_Report_(Original_Submission) -- Thomas Hackl — 04/20/2018 Reviewed [file giy075_reviewer_2_report_(original_submission).pdf]

Reviewer Report

Title: **Binning Enables Efficient Host Genome Reconstruction in Cnidarian Holobionts**

Version: **Original Submission** **Date:** 4/20/2018

Reviewer name: **Thomas Hackl**

Reviewer Comments to Author:

Celis et al. describe how the genome assembly of a stony coral can be obtained from a sequencing sample of the coral holobiont, also containing symbiont and microbial DNA. The authors first assemble the sample as a metagenome data set, and identify contigs of the eukaryotic host using contig binning techniques designed for microbial metagenomics. Then reads mapped to the host contigs are extracted and reassembled to generate a host-only genome assembly. The authors further evaluate the assembly and show that it meets community standards, and in important aspects is on par with published assemblies of other cnidarians obtained from symbiont-free samples.

I believe that the approach presented by Celis et al. is robust, a good way to overcome the need for tissue-free samples for cnidarians and potentially a range of other organisms, and thus of great value to a broader community. Moreover, the manuscript is well written, and I do not have any issues to report.

A short list of non-issues for which I leave it at the author's discretion whether or not to address them.

I have not had any experience with gsAssembler, and I do not want to get in the "what is the best assembler" discussion, but I'd be curious as to why you chose gsAssembler. I would not have been my first choice (and similar for Ray Meta). My guess is that you could get better results with a combo: metaSPAdes + dipSPAdes.

Have you thought about exploring reads not mapped, or mapping to unbinned contigs in combination with a gap-filling tool or similar to improve contiguity of the assembly. I could see a lot of the assembly gaps being related to reads not mapped to the contigs in your 13 host bins.

Binning is quite sensitive to contig length. I think that this approach could be even more powerful when used in combination with PacBio/Nanopore sequencing. You would get much more long contigs in the initial assembly, therefore better binning, and in turn higher completeness and, in particular, higher contiguity in the host-only assembly.

Methods

Are the methods appropriate to the aims of the study, are they well described, and are necessary controls included? Choose an item.

Conclusions

Are the conclusions adequately supported by the data shown? Choose an item.

Reporting Standards

Does the manuscript adhere to the journal's guidelines on minimum standards of reporting? Choose an item.

Choose an item.

Statistics

Are you able to assess all statistics in the manuscript, including the appropriateness of statistical tests used? Choose an item.

Quality of Written English

Please indicate the quality of language in the manuscript: Choose an item.

Declaration of Competing Interests

Please complete a declaration of competing interests, considering the following questions:

- Have you in the past five years received reimbursements, fees, funding, or salary from an organisation that may in any way gain or lose financially from the publication of this manuscript, either now or in the future?
- Do you hold any stocks or shares in an organisation that may in any way gain or lose financially from the publication of this manuscript, either now or in the future?
- Do you hold or are you currently applying for any patents relating to the content of the manuscript?
- Have you received reimbursements, fees, funding, or salary from an organization that holds or has applied for patents relating to the content of the manuscript?
- Do you have any other financial competing interests?
- Do you have any non-financial competing interests in relation to this paper?

If you can answer no to all of the above, write 'I declare that I have no competing interests' below. If your reply is yes to any, please give details below.

I agree to the open peer review policy of the journal. I understand that my name will be included on my report to the authors and, if the manuscript is accepted for publication, my named report including any attachments I upload will be posted on the website along with the authors' responses. I agree for my report to be made available under an Open Access Creative Commons CC-BY license (http://creativecommons.org/licenses/by/4.0/). I understand that any comments which I do not wish to be included in my named report can be included as confidential comments to the editors, which will not be published.

I agree to the open peer review policy of the journal

To further support our reviewers, we have joined with Publons, where you can gain additional credit to further highlight your hard work (see: https://publons.com/journal/530/gigascience). On publication of this paper, your review will be automatically added to Publons, you can then choose whether or not to claim your Publons credit. I understand this statement.

Yes Choose an item.
